# Investigating Lightweight Carbonation Curing of Waste Slurry Using Activated Magnesium Oxide: Performance Insights

**DOI:** 10.3390/ma18092084

**Published:** 2025-05-01

**Authors:** Li Shao, Wangcheng Yu, Xi Du, Aiping Shen, Yi Li, Huihong Ding, Shichao Tao, Fangjin Wu

**Affiliations:** 1 School of Environment and Architecture, University of Shanghai for Science and Technology, Shanghai 200093, China; shaoli1999@usst.edu.cn (L.S.);; 2 USST Center for Instrumental Analysis, University of Shanghai for Science and Technology, 516 Jungong Road, Shanghai 200093, China; shenaiping@usst.edu.cn; 3 Department of Engineering Geology, Institute of Applied Geosciences, Technical University of Berlin, Ernst-Reuter-Platz 1, BH 3-1, 10587 Berlin, Germany; 4 School of Transportation, Southeast University, No. 2, Southeast University Road, Jiangning District, Nanjing 211189, China; 5 School of Civil Engineering, Kashi University, Kashi 844008, China

**Keywords:** activated magnesium oxide, calcium carbide slag, mineral powder, sustainable materials, environmental impact

## Abstract

This study, from the perspective of resource utilization and carbon sequestration, developed a novel lightweight carbonated solidified slurry material using reactive magnesium oxide (MgO), ground granulated blast furnace slag (GGBS), and carbide slag as stabilizers, with carbonation induced by a CO_2_ foaming method. The physical and mechanical properties of the material were investigated. Based on the optimal mix proportion, the effects of MgO content, CO_2_ foam dosage, and stabilizer dosage on wet density, flowability, moisture content, and unconfined compressive strength were analyzed. The results indicate that wet density increases with increasing MgO and stabilizer content but decreases with increasing CO_2_ foam dosage. Flowability decreases with increasing MgO and stabilizer content but improves with a higher CO_2_ foam dosage. Unconfined compressive strength increases with curing age and stabilizer content but decreases with increasing CO_2_ foam dosage. Additionally, prolonged curing enhances both moisture content and unconfined compressive strength. These findings provide a theoretical basis for engineering applications.

## 1. Introduction

Heavy metal pollution from waste mud is a global issue impacting numerous countries, primarily resulting from activities such as mining, mineral processing, the discharge of agricultural wastes, unscientific use of fertilizers and pesticides, and sewage irrigation [1]. The accumulation of heavy metals in agricultural soils poses serious ecological hazards and food safety concerns due to their toxicity [2]. Even at low concentrations, highly toxic heavy metals present significant ecological risks to soil and water environments, with many capable of inducing cancer in humans [3,4,5,6]. To mitigate the severe harm caused by soil heavy metal pollution, various remediation technologies have been developed and implemented over time.

Concurrently, the advancement of urbanization and heightened environmental awareness have brought global warming—caused by the greenhouse effect—into focus. Reducing carbon dioxide emissions, the primary greenhouse gas, and managing its disposal have become critical environmental challenges. Research indicates that capturing and storing carbon dioxide is the most direct and effective method to decrease its atmospheric concentration. Current strategies for controlling carbon dioxide levels include enhancing its utilization rate, reducing emissions at the source, geological storage, oceanic storage, and mineral carbonation [7]. In the field of civil engineering, the introduction of carbon sequestration theories and technologies offers innovative approaches for developing “green civil engineering”.

Waste slurry treatment technologies primarily comprise natural sedimentation, slurry flocculation and dewatering, and slurry curing techniques. Among these, slurry curing is recognized as the most ideal and widely adopted method for handling waste mud. This technique involves adding a curing agent to the waste mud, initiating a series of physical and chemical reactions that achieve dewatering and flocculation. Post-curing, the mud exhibits high strength, good fluidity, and low cost, making it suitable for applications like roadbed filling.

Portland cement is the most commonly used binder, but its production consumes significant resources and energy, leading to environmental impacts. To develop low-carbon, environmentally friendly, and cost-effective reinforcement methods and solidification materials, researchers worldwide have extensively studied alternative cementitious materials. In recent years, replacing cement partially or entirely with industrial by-products or waste materials has become a research focus. In the early 21st century, Australian scientist Harrison [8,9,10] developed a novel low-carbon cement composed of reactive magnesium oxide (MgO) and Portland cement. This material offers distinct advantages in energy efficiency, environmental sustainability, and mechanical performance.

Reactive MgO is typically produced by calcining magnesite at approximately 700 °C, which is only half the clinker sintering temperature of cement (>1450 °C). This process reduces both energy consumption and CO_2_ emissions. Furthermore, during carbonation, reactive MgO absorbs a substantial amount of CO_2_, making it a more sustainable binder than cement [10]. Proven reserves of magnesite are approximately 13 billion tons, with several million tons of brucite deposits. The magnesium content in seawater is estimated at around 6 × 10^16^ tons. Additionally, significant dolomite and salt lake magnesium resources are available. China possesses the world’s largest magnesium resources, accounting for 22.5% of global reserves. The country’s proven magnesite reserves total 3.4 billion tons, representing 28.3% of the global supply, ranking first worldwide. Additionally, China’s dolomite reserves exceed 4 billion tons. The four major salt lakes in the Qaidam Basin, including brine lakes, semi-dried salt lakes, and dried salt lakes, contain an estimated 6.003 billion tons of magnesium salts [11].

Since 2004, the A1-Tabbaa research group at the University of Cambridge’s Department of Engineering has conducted systematic studies on reactive MgO cement, including its carbonation performance [12], hydration characteristics [13], and microstructure [14], while also promoting its commercialization. Reactive MgO cement is produced by blending Portland cement (PC) with MgO in varying proportions, offering potential sustainability and technical advantages [15]. Studies by Vandeperre et al. [12,14] and Liska [16] indicate that the hydration of PC and reactive MgO in MgO-PC mixtures occurs independently, forming calcium silicate hydrate (CSH) and low-strength Mg(OH)_2_, respectively. Consequently, the contribution of MgO to strength enhancement is limited, and at higher MgO dosages, it may even lead to strength reduction. In 2010, Southeast University and the University of Cambridge jointly proposed the use of the MgO carbonation deep mixing method for soft ground improvement and applied for related patents [17,18]. The carbonation deep mixing method is considered a novel low-carbon ground improvement technique. In this method, reactive MgO is used as a soil stabilizer and thoroughly mixed with the soil. CO_2_ is then introduced into the mixture for several hours, triggering intense hydration and carbonation reactions between MgO, water, and CO_2_, leading to the formation of magnesium carbonates. This process significantly reduces soil moisture content and porosity while enhancing strength. Additionally, the method absorbs a substantial amount of CO_2_ during stabilization, making it an environmentally friendly, low-carbon solution.

Existing research primarily focuses on the carbonation curing of soft soil using activated magnesium oxide and the preparation of foam lightweight soil using traditional cement curing agents. However, there is a lack of in-depth research on the curing technology of high-water-content waste mud, the use of new green curing agents for preparing foam lightweight soil, and the foaming process of foam lightweight soil.

Based on the current research on waste slurry solidification, Wu [19] selected the contents of ground granulated blast furnace slag (GGBS), reactive magnesium oxide (MgO), and carbide slag as three key factors for a three-factor, three-level orthogonal experiment. The physical and mechanical properties of lightweight carbonated solidified slurry were evaluated using flowability, wet density, and 28-day unconfined compressive strength as performance indicators. Through range analysis, the optimal baseline mix proportion under different working conditions was determined as follows: 25% GGBS, 12.5% reactive MgO, and 7.5% carbide slag.

With the optimal mix ratio established, we measured wet weight, fluidity, moisture content, and unconfined compressive strength at different curing ages to investigate the influence of active magnesium oxide content, CO_2_ foam content, and curing agent content on the physical and mechanical properties of the lightweight carbonized cured slurry. We analyzed the effects of different factors on performance to provide a theoretical basis for engineering applications.

Therefore, the main objective of this study is to develop and evaluate a novel lightweight carbonated cured slurry system using reactive magnesium oxide, industrial by-products, and CO_2_ foam, targeting high-water-content waste slurries in geotechnical applications. The greatest achievements of this work include (1) establishing an optimal composite curing agent formulation that balances strength, flowability, and ductility; (2) demonstrating CO_2_ foam as an effective functional filler to improve flowability and reduce density; and (3) proposing a sustainable, carbon-utilizing stabilization strategy using low-carbon binders and industrial residues. These findings offer a valuable reference for the development of new low-carbon cementitious materials and promote the application of carbonation curing technology in the field of soil stabilization and lightweight concrete.

## 2. Technical Programs

### 2.1. Test Materials and Program

#### 2.1.1. Test Materials

(1) Waste slurry

**Figure 1:**
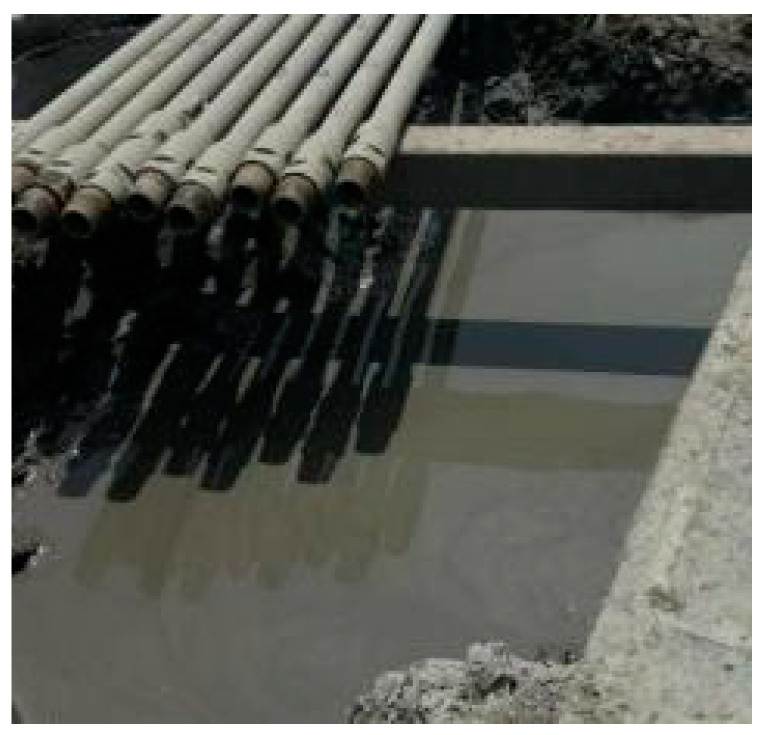
Construction site waste slurry.

**Figure 2:**
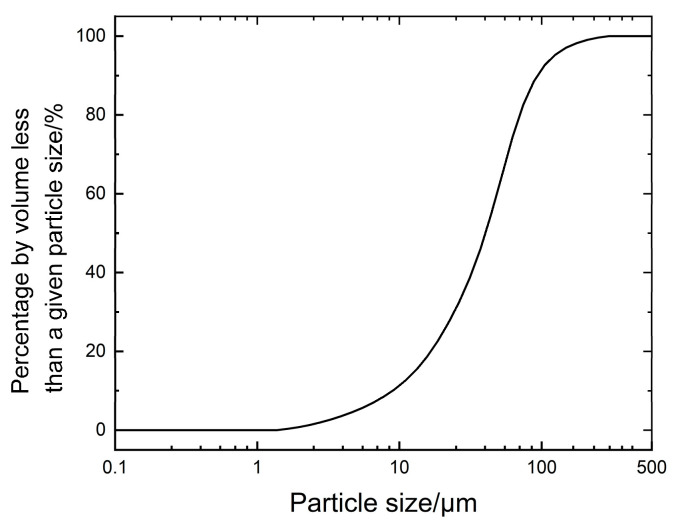
Particle analysis curve for waste slurry.

The waste mud used in this study was collected from the drilled pile mud at a construction site in West Coast Camp, Xuhui District, Shanghai. As shown in Figure 1, the mud was gray-black in color and had a high water content. Sampling, transportation, and storage were conducted in sealed drums to prevent water evaporation. The water content, pH, liquid limit, plastic limit, and specific gravity were determined according to the *Standard for Geotechnical Test Methods* (GBT50123-2019) [20] and the *Test Procedure for Fixed-wall Mud of Hydropower and Water Conservancy Engineering* (DL/T5815-2020) [21]. The specific physical indices are presented in Table 1. Additionally, the soil samples were subjected to particle size distribution analysis using a laser particle sizer, and the results are shown in Figure 2.

Figure 2 shows that the particle sizes of the waste slurry are mainly concentrated between 10 and 100 µm, indicating a narrow particle size distribution and poor gradation.

(2) Magnesium oxide

The STARMAG150 active magnesium oxide used in this test was chemically extracted from seawater, and the manufacturer was Kamishima, Japan. It appears as a pure white, ultrafine powder with a density of 0.48 g/cm^3^ and a magnesium oxide content of up to 98%. Its chemical composition and content are shown in Table 2. The material exhibits excellent reactivity and high densification.

(3) Calcium carbide slag

This test selected the quality grade for high-quality first-class calcium carbide slag powder, which is a fine, gray powder with high activity, a particle size of 150–400 mesh or so, and a density of 1.5 g/cm^3^; the specific chemical composition is shown in Table 3.

(4) Mineral powder

For this test, S95-grade slag powder was selected, characterized by a density of 2.8 g/cm^3^, a flow ratio of 98%, a loss on ignition of 0.84%, and a moisture content of 0.45%. It is gray-white in appearance, and its specific chemical composition is provided in Table 4. The activity index of the mineral powder is a crucial indicator for assessing its quality. It is determined by preparing mortar test blocks using cement and mineral powder in a mass ratio of 1:1 and testing their compressive strength at 7 and 28 days. These results are compared with those of pure cement mortar test blocks at the same ages. In this study, the selected S95-grade mineral powder has a 28-day activity index of 98.5%. An activity index exceeding 95% indicates that the compressive strength of mortar containing 50% mineral powder is at least 95% of that of pure cement mortar over the same curing period.

(5) Anhydrous calcium sulfate, PAM, and CO_2_

The anhydrous calcium sulfate used in this test was of analytically pure AR quality and the specific technical conditions are shown in Table 5.

Polyacrylamide (anionic) is analytically pure, also known as PAM; its PAM content is no less than 90%, with a hydrolysis degree of 30%, white crystals, a pH value of 5.0–7.0, and anionic value of 1.2–1.6, and it has a good flocculation effect. CO_2_ gas is high-purity food-grade CO_2_ with 99% purity.

(6) Foaming agents

**Figure 3:**
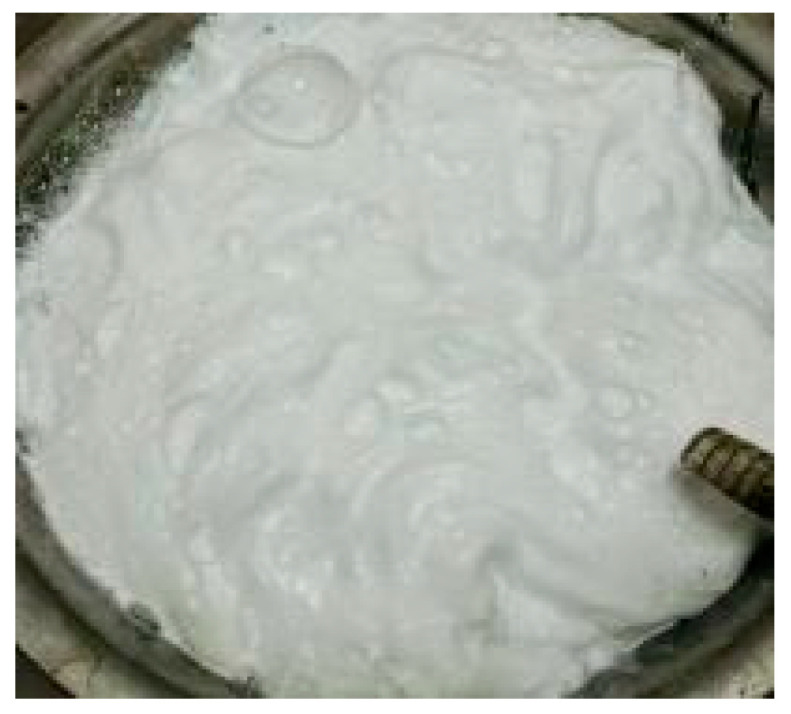
CO_2_ foam used in the test.

Currently, foaming agents are classified into four types: rosin resin-based, animal and plant protein-based, surfactant-based, and composite types. In this test, a composite foaming agent was used, which produces higher foam expansion ratios and better foam stability. When air is used for foaming, the density of the air foam is 40 kg/m^3^; when CO_2_ is used for foaming, the density of the CO_2_ foam is 80 kg/m^3^. An image of the CO_2_ foam is shown in Figure 3.

#### 2.1.2. Foam Dosage and Mix Design Calculations

Based on the optimal mix proportion, this study investigates the effects of reactive magnesium oxide (MgO) content, CO_2_ foam dosage, and stabilizer dosage on the physical and mechanical properties of lightweight carbonated solidified slurry. Experiments were conducted to measure wet density, flowability, and moisture content, as well as unconfined compressive strength at curing ages of 7, 28, 60, and 90 days. Calcium carbide slag, serving as an alkali activator, provides an alkaline environment to stimulate the activity of the mineral powder and activated magnesium oxide. Since high-water-content waste slurry without mud–water separation treatment was used, anhydrous calcium sulfate and polyacrylamide (PAM) were added as admixtures to reduce water content and achieve better curing effects. Based on the test results of Zhou et al. [22] and Yang [23], the dosages of anhydrous calcium sulfate and PAM were set at 5% and 0.5% of the total mass of the waste slurry, respectively. The stabilizer dosage refers to the sum of the contents of ground granulated blast furnace slag (GGBS), reactive magnesium oxide (MgO), and carbide slag, with the proportions of these components derived by scaling down the ratios obtained from Wu Fangjin’s orthogonal experiment. Both the stabilizer dosage and the dosages of other solid powders are expressed as percentages of the total mass of the waste slurry.

The CO_2_ foam dosage is defined as the percentage of the total volume of CO_2_ foam added to the total volume of the lightweight carbonated cured slurry. According to current research [24], this study selected a CO_2_ foam dosage of 35% of the total volume. The specific proportion calculation method refers to the “*Technical Specifications for Filling Engineering with Bubble Mixed Lightweight Soil*” (CJJ/T177-2012) [25]. With a foam expansion ratio of 40 times and a CO_2_ foam density of 80 kg/m^3^.

The calculation of CO_2_ foam content in the lightweight carbonated cured slurry is based on the volume balance and mass–density relationship of the mixture. According to the *Technical Specification for Filling Engineering with Bubble Mixed Lightweight Soil* (CJJ/T 177–2012) [25], the total unit mass of the slurry is determined by summing the contributions of all solid components and the foam volume. The CO_2_ foam dosage, defined as the volume percentage of foam in the total slurry mixture, the proportion calculation is carried out according to the following formula:

(1) $$\frac{m_{m}}{\rho_{m}}+\frac{m_{n}}{\rho_{n}}+\frac{m_{k}}{\rho_{k}}+\frac{m_{d}}{\rho_{d}}+\frac{m_{s}}{\rho_{s}}+\frac{m_{f}}{\rho_{f}}=1$$

(2) $$m_{m}+m_{n}+m_{k}+m_{d}+m_{s}+m_{f}=100p$$

where *m_m_*: mass of activated magnesium oxide per cubic meter of bubble-mixed lightweight soil (kg);

*ρ_m_*: density of active magnesium oxide (kg/m^3^), take 480 kg/m^3^;

*m_n_*: mass of waste slurry per cubic meter of bubble-mixed lightweight soil (kg);

*ρ_n_*: density of waste slurry (kg/m^3^), take 1180 kg/m^3^;

*m_k_*: mass of mineral powder (kg) used in each cubic meter of bubble-mixed lightweight soil;

*ρ_k_*: mineral powder density (kg/m^3^), take 2800 kg/m^3^;

*m_d_*: mass of calcium carbide slag (kg) used in each cubic meter of bubble-mixed lightweight soil;

*ρ_d_*: density of calcium carbide slag (kg/m^3^), take 1500 kg/m^3^;

*m_s_*: mass of anhydrous calcium sulfate per cubic meter of air bubble mixed lightweight soil (kg);

*ρ_s_*: density of anhydrous calcium sulfate (kg/m^3^), take 2600 kg/m^3^;

*m_f_*: mass of CO_2_ bubbles per cubic meter of bubble-mixed lightweight soil (kg);

*ρ_f_*: CO_2_ bubble density (kg/m^3^), take 80 kg/m^3^;

*p*: bubble-mixed lightweight soil foam rate (%).

The specific test design table is shown in Table 6, Table 7 and Table 8.

Equation (1) derives the total solid mass per unit volume based on the densities and dosages of each component, while Equation (2) relates the foam mass to its volume and density. These equations assume the following: (1) The foam is uniformly distributed throughout the slurry; (2) the density of each component remains constant during mixing; (3) the total volume is the sum of the component volumes (additive volume assumption); and (4) no significant air leakage or foam collapse occurs before mixing.

The mass of each ingredient is converted to volume using known densities, allowing foam content (as a volume ratio) to be calculated. The equations are adapted from CJJ/T 177–2012 and commonly used in bubble-mixed lightweight soil engineering.

### 2.2. Specimen Preparation and Test Content

#### 2.2.1. Specimen Preparation and Carbonization

**Figure 4:**
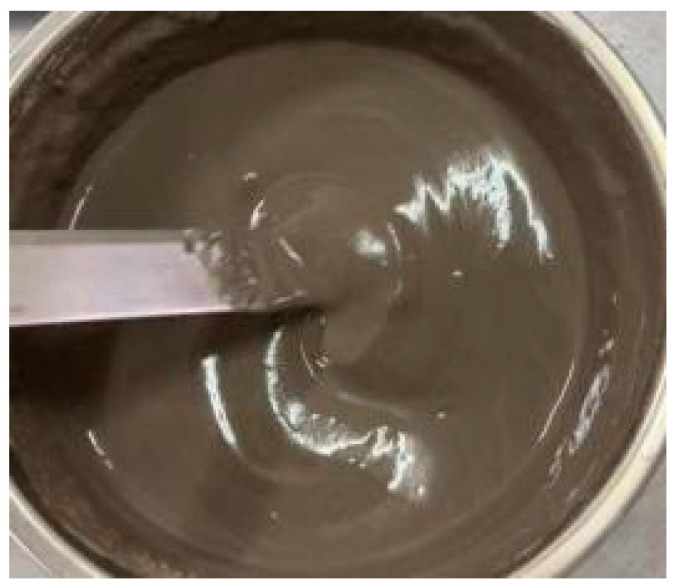
Slurry after curing with curing agent.

The slurry collected from the construction site was pre-treated by mixing and drying, and then stored in a sealed container. Before testing, the slurry was adjusted to a preset water content of 200%. The curing agent was weighed according to the calculated ratios. The curing agent powder was first thoroughly mixed and then slowly added to the high-water-content slurry in two batches, stirring for 3 min each time to ensure complete mixing of the curing agent and slurry. As shown in Figure 4, the cured slurry exhibits a more consolidated and structured form compared to its untreated state, demonstrating the initial solidification effect of the curing agent on high-water-content waste mud.

**Figure 5:**
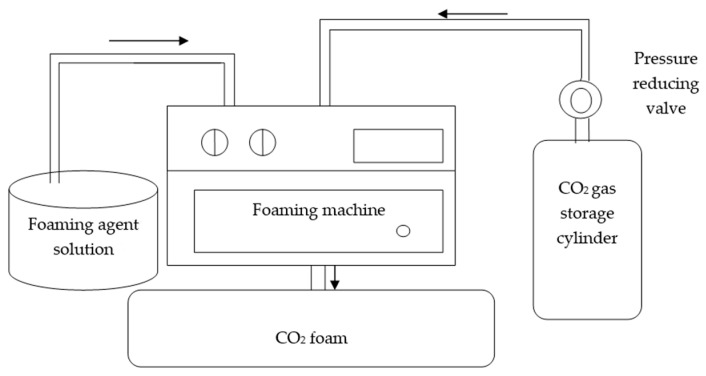
Schematic diagram of the foaming machine. (The arrows in the diagram refer to the flow of gases, liquids, and foams.).

The complete configuration and working principle of the foaming system are illustrated in Figure 5, which outlines the foaming agent reservoir, CO_2_ source, pressure regulation system, and foam delivery unit. To perform CO_2_ foaming, the foaming agent must first be pre-treated. Because the foaming agent is alkaline and readily reacts with CO_2_—which is somewhat soluble in the solution—this can result in unstable foam that defoams quickly. Therefore, pre-treatment is necessary. Dilute the foaming agent at a ratio of 1:40 and then introduce CO_2_ gas and adjust the pressure to 200 kPa. Wait until the foaming stabilizes and then close the gas valve and let the mixture stand for 20 min to allow the CO_2_ and foaming agent to fully react. During this period, maintain stable gas pressure in the foaming machine; if the pressure drops, reopen the valve to introduce more CO_2_ and stabilize the pressure. Next, turn on the foaming machine and adjust the foaming pressure. Once stable foam is produced, let it stand for 10 min to achieve uniformity and stability.

Since the lightweight carbonated curing slurry is alkaline, using an acidic blowing agent would cause the resulting CO_2_ foam to easily dissolve in the slurry, leading to significant defoaming and collapse, which is detrimental to strength development. Therefore, this study chooses an alkaline blowing agent. Although it readily reacts with CO_2_, pre-treatment can mitigate this effect. Using an alkaline blowing agent ensures that the CO_2_ foam is insoluble in the lightweight carbonated curing mud, preventing noticeable collapse and having minimal impact on the material’s strength.

The resting foam is prepared according to the ratio with a measuring cylinder. A good mixing slurry is added, and stirring is continued for 1 min so that the foam and slurry are fully mixed uniformly for carbonation, and then the mixed slurry is poured into the mold, which is used three times throughout the test; due to the fact that the foam lightweight soil has self-tightening properties, a slight shock means the mold can be filled uniformly. Finally, after pouring, the mold is covered with a transparent cling film to prevent moisture dissipation.

The poured specimen was placed in a standard curing box at a temperature of 20 °C (±1 °C) and a humidity of 99% for standard curing and was molded after 48 h, and the molded specimen was covered with cling film and placed in the curing box to continue standard curing until the required age of the test.

**Figure 6:**
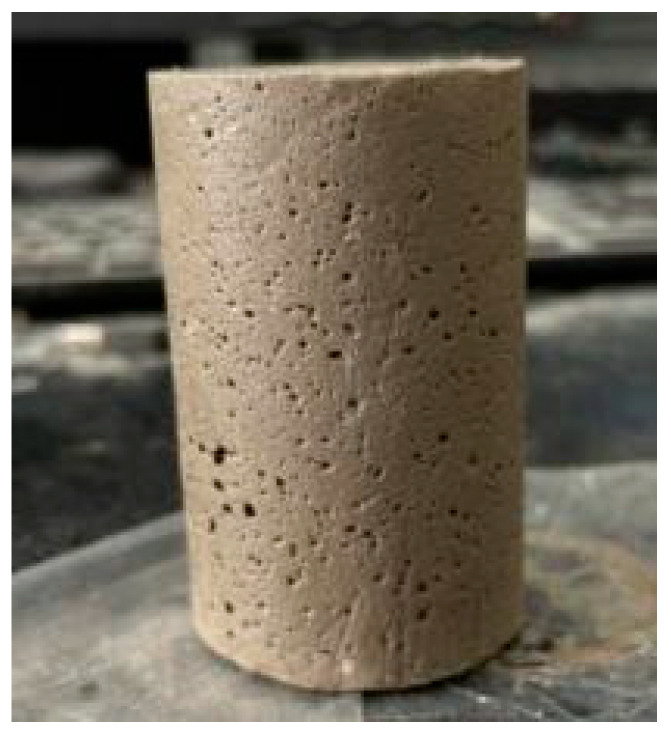
Specimen after demolding.

As depicted in Figure 6, the specimens maintain their structural integrity after demolding, indicating good cohesion and sufficient early-age strength, which is essential for subsequent curing and mechanical testing.

#### 2.2.2. Test Content and Methodology

The mixed slurry was subjected to a wet weight test according to the *Technical Specification for Filling Engineering with Bubble Mixed Lightweight Soil* (CJJ/T177-2012) [25]. Wet weight, also known as wet bulk weight, is the mass per unit volume of the slurry before curing. To perform the test, the slurry was poured into a volumetric cylinder after being shaken and scraped with a spatula to ensure uniformity. The filled cylinder was then weighed with an accuracy of 1 g. After weighing, the slurry was removed, and the cylinder was cleaned. This procedure was repeated three times, and the arithmetic mean of the three measurements was taken as the final test result. It is important to conduct the wet weight test within 5 min after mixing the slurry, and the slurry should not be reused to prevent curing, which could lead to inaccurate results. The wet weight was calculated using Equation (3):

(3) $$\gamma =\frac{10\times \left(m_{1}-m_{0}\right)}{v_{0}}$$

Note: the value of gravitational acceleration is taken as 10 m/s^2^ according to the engineering standard *CJJ/T 177-2012*, which specifies this approximation for use in practical calculations of unit weight in foamed lightweight soil applications.

Here, γ: specimen wet weight (kN/m^3^), accurate to 0.1 kN/m^3^;

*m*_1_: total mass of specimen and volumetric cylinder (g), accurate to 0.1 g;

*m*_0_: mass of the volumetric cylinder (g), accurate to 0.1 g;

*v*_0_: volume of the volumetric cylinder (cm^3^), accurate to 0.1 cm^3^.

The flowability of the specimen was tested according to the *Technical Specification for Bubble Mixed Lightweight Soil Filling Engineering* (CJJ/T177-2012) [25]. Flowability, also known as the flow value, is used to assess the slurry’s flow performance. According to the specification, the flow degree of bubble-mixed lightweight soil generally ranges between 160 and 200 mm.

To conduct the test, a polished ceramic tile and a foam lightweight soil fluidity test cylinder (inner diameter of 80 mm, height of 80 mm, and wall thickness of 2 mm) were prepared and placed horizontally. The stirred slurry was slowly poured into the cylinder, ensuring it did not spill outside, and the surface was leveled with a flat spatula. The cylinder was then carefully lifted vertically until all the slurry flowed out, forming a disk shape on the ceramic tile. After 1 min, the diameter of the disk-shaped slurry was measured using vernier calipers accurate to 1 mm and recorded. This procedure was repeated three times, and the arithmetic mean of the three measurements was taken as the flow value of the slurry.

The unconfined compressive strength of the specimen was tested according to the *Specification for Geotechnical Testing of Highways* (JTG3430-2020) [26]. The mixed slurry was poured into cylindrical molds with a diameter of 50 mm and a height of 100 mm. After 48 h of standard curing at 20 °C and 99% humidity, the specimens were demolded and continued curing under the same conditions for a total of 28 days.

**Figure 7:**
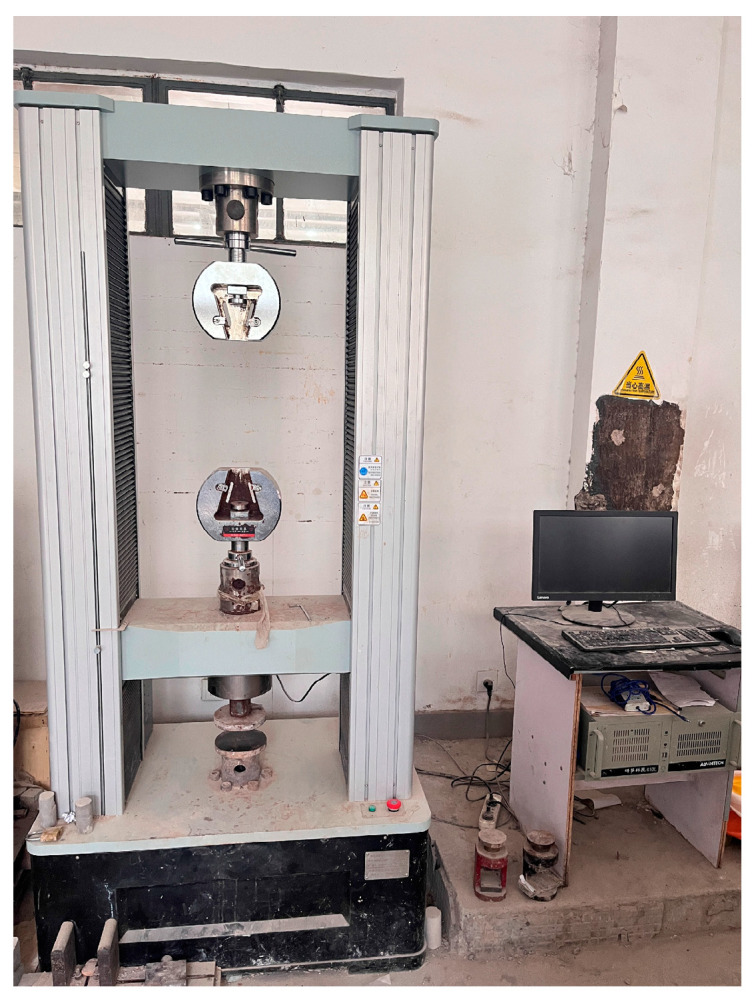
Press used for unconfined compressive strength.

Figure 7 shows the press used for unconfined compressive strength. For the test, place the cured specimen on the base of the compression testing machine. Set the displacement rate of the press to 1 mm/min. Conduct the unconfined compressive strength test until the load reaches a peak or the readings stabilize. Then, continue loading to achieve an additional axial strain of 3–5% before stopping the test. If the readings do not stabilize, continue the test until the axial strain reaches 20%, ensuring that the total test duration is between 8 and 10 min. Prepare three parallel specimens and repeat the test for each. Calculate the arithmetic mean of the three test results to determine the unconfined compressive strength value. The “breaking strain” is defined as the axial strain corresponding to the peak compressive stress during the unconfined compressive strength test. All strain values were recorded from the point of first load contact (zero-load alignment) to the failure point.

Calculate the unconfined compressive strength according to the following formula:

(4) $$\varepsilon =\frac{\Delta h}{h_{0}}$$

(5) $$\sigma =\frac{P}{A_{a}}=\frac{P}{A_{0}/\left(1-\varepsilon \right)}$$

Equation (5) is used to characterize the strain energy density based on axial stress–strain behavior under unconfined compressive loading. Since the test setup involved uniaxial loading and only axial strain was recorded, the influence of lateral (transverse) deformation was not considered. For simplification, Poisson’s ratio was omitted, and the energy calculation is based solely on axial deformation. This approach is commonly used in studies focusing on compressive toughness or brittleness indices where lateral strain data are not available.

Here, *ε*: axial strain (%);

*h*_0_: initial height of the test block (cm);

Δ*h*: axial deformation (cm);

*A_a_*: cut-off area of the corrected specimen (cm^2^);

*A*_0_: initial cross-sectional area of the test block (cm^2^);

*P*: load at destruction of test block (N);

*σ*: unconfined compressive strength (kPa).

Carbonation and curing of the waste slurry were performed according to the designed test ratios, following the same specific methods as previously described. For the unconfined compressive strength tests, molds made of ABS engineering plastic with a diameter of 50 mm and a height of 100 mm were used, in accordance with the *Specification for Geotechnical Testing of Highways* (JTG3430-2020).

The specimens for water content testing were the remnants after conducting the unconfined compressive strength tests at a curing age of 28 days. Approximately 20 g of the destructed specimens was collected, further broken down, and then passed through a 2 mm sieve. The mass was recorded, and the test was initiated within 5 min after the unconfined compressive strength test to prevent evaporation, which could lead to inaccurate data. The samples were placed in aluminum containers and dried in a blast drying oven at 40 °C. It is important to maintain the drying temperature below 50 °C because higher temperatures may dehydrate magnesite trihydrate formed during carbonation, converting it to alkaline magnesium carbonate [27]. The drying process was monitored every day for 2 days until the mass of the dried mud remained constant, and the final mass was recorded. Three parallel tests were conducted for each group, and the arithmetic mean of the three results was taken as the water content of the specimens.

The moisture content of the specimen is calculated according to the following formula:

(6) $$w=\frac{m-m_{s}}{m_{s}}$$

where *w*: moisture content of the specimen (%), accurate to 0.1%;

*m*: undried mass of the specimen (g);

*m_s_*: mass of the specimen after drying (g).

## 3. Analyses

Considering the physical and mechanical properties, particularly the 28-day unconfined compressive strength, the optimal mix ratio of the lightweight carbonated cured slurry was determined to be 25% mineral powder, 12.5% activated magnesium oxide, and 7.5% calcium carbide slag. This ratio yields the highest 28-day compressive strength, which has a significant positive impact on tunnel excavation and backfilling, improving overall safety and stability.

### 3.1. Effect of Active Magnesium Oxide Dosage

In this paper, we used waste mud as the research material and conducted experimental studies by adding active magnesium oxide at dosages of 7%, 10%, 13%, 16%, 19%, and 21% of the total mass of the waste mud. By analyzing how different dosages affected wet weight, fluidity, moisture content, unconfined compressive strength, and other physical and mechanical properties, we investigated the impact of active magnesium oxide on the carbonation and curing effects of waste slurry, thereby determining the optimal dosage of active magnesium oxide.

#### 3.1.1. Effect of Active Magnesium Oxide Dosage on the Wet Weight of Lightweight Carbonated Cured Mud

**Figure 8:**
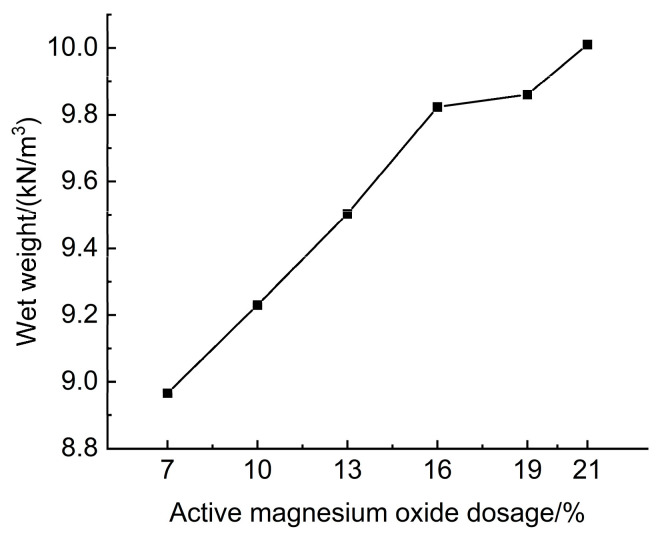
Effect of active magnesium oxide dosing on wet weight.

The experimental results of the effect of active magnesium oxide dosing on the wet weight of lightweight carbonated cured mud are shown in Figure 8.

Figure 8 illustrates the impact of activated magnesium oxide dosage on the wet weight of lightweight carbonized cured mud. The data indicate that the wet weight of the lightweight carbonation-cured mud ranges from 8.96 to 10.01 kN/m^3^, with an increase in wet weight corresponding to a higher dosage of activated magnesium oxide. The hydration reaction between MgO and water produces Mg(OH)_2_, which rapidly engages in a series of carbonation reactions with CO_2_ foam, ultimately yielding magnesium carbonate compounds, such as magnesia stone. The specific reaction formula is as follows:

(7) $$M_{g}O+H_{2}O\to M_{g}{\left(OH\right)}_{2}$$

(8) $$M_{g}{\left(OH\right)}_{2}+{CO}_{2}+2H_{2}O\to M_{g}{CO}_{3}\cdot 3H_{2}O$$

Formulas (7) and (8) are derived from the stoichiometric conversion of MgO and CaO to their respective carbonates, assuming complete carbonation reactions. These theoretical calculations provide a useful estimate of potential CO_2_ sequestration. However, no direct chemical analyses (e.g., thermogravimetric analysis or X-ray diffraction) were performed in this study to confirm the extent or completeness of carbonation. Future work will incorporate such techniques to better quantify the actual carbonate content and validate these assumptions.

The hydration and carbonation products of the lightweight carbonized cured mud increased with higher dosages of activated magnesium oxide, resulting in a greater wet weight. At an activated magnesium oxide dosage of 21%, the wet weight of the lightweight carbonation-cured mud reached a maximum of 10.01 kN/m^3^. This study utilized a mix ratio consisting of 13% activated magnesium oxide, 7.5% calcium carbide slag, 25% mineral powder, 0.5% polyacrylamide (PAM), and 5% anhydrous calcium sulfate. Tests revealed that without the CO_2_ foaming method, the wet weight of the curing mud was 15.16 kN/m^3^, significantly higher than that of the lightweight carbonation-cured mud. This indicates that the lightweight carbonation-cured mud has a reduced self-weight, effectively achieving the goal of lightweight construction.

#### 3.1.2. Effect of Active Magnesium Oxide Dosage on Flowability

**Figure 9:**
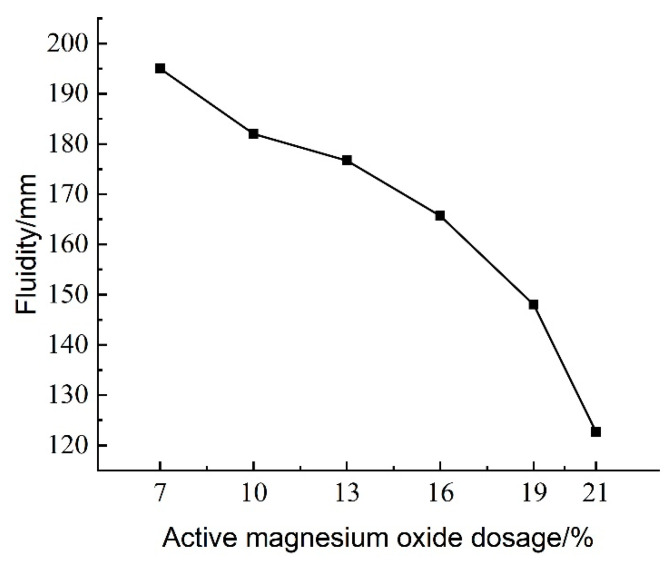
Effect of active magnesium oxide dosing on flowability.

The experimental results of the effect of active magnesium oxide dosing on the flowability of lightweight carbonated cured mud are shown in Figure 9.

**Figure 10:**
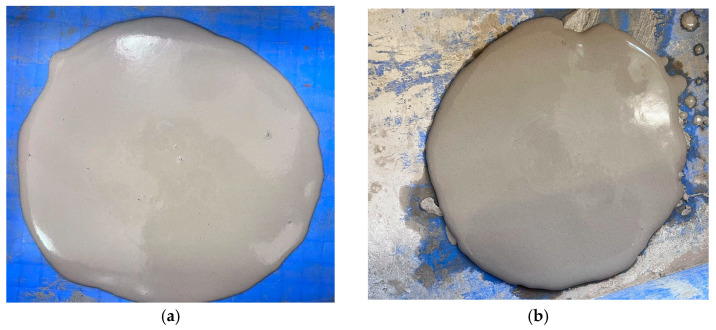
Representative flow test results for slurry with extreme fluidity values. (**a**) Maximum flowability; (**b**) minimum flowability.

Figure 9 illustrates the effect of activated magnesium oxide dosing on the flow characteristics of the lightweight carbonation-cured slurry. The flow values, as shown in Figure 9, range from 122 to 195 mm. Notably, the flow decreases with increasing activated magnesium oxide dosage. At a dosage of 7%, the slurry exhibits the highest flow rate of 195 mm; beyond this point, the flow rate declines sharply as the dosage continues to increase. Figure 10a Maximum flowability; Figure 10b minimum flowability.

The hydration reaction between activated magnesium oxide and the high water content in the waste slurry produces Mg(OH)_2_, which further reacts with CO_2_ foam to form solid crystalline carbonation products. As the dosage of activated magnesium oxide increases, the hydration reaction within the waste slurry intensifies, leading to greater water consumption and a corresponding decrease in water content. Simultaneously, the formation of solid crystalline carbonation products increases, resulting in diminished fluidity of the lightweight carbonation-cured mud. When the waste slurry is not treated to separate the mud from the water during carbonation curing, it remains in a liquid form that lacks shape retention. However, the addition of curing agents and optimization of the CO_2_ foaming process can enhance the fluidity of the high-water-content waste slurry, allowing it to be poured directly without separation.

#### 3.1.3. Effect of Active Magnesium Oxide Dosing on Water Content

**Figure 11:**
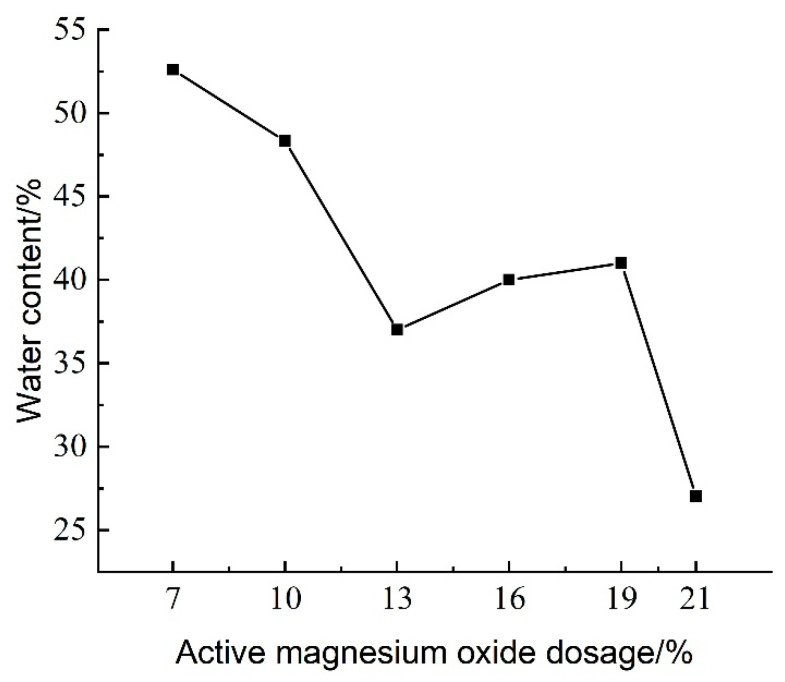
Effect of active magnesium oxide dosing on water content.

The effect of active magnesium oxide dosing on the water content of lightweight carbonated cured mud at a curing age of 28 d is shown in Figure 11.

Figure 11 illustrates the effect of activated magnesium oxide dosing on the water content of lightweight carbonized cured slurry. As shown in the figure, the dosage of activated magnesium oxide significantly influences the water content, which initially decreases, then increases, and subsequently decreases again with increasing magnesium oxide levels. The minimum water content, recorded at 21% magnesium oxide dosing, is 27.03%.

In the waste mud, water undergoes a hydration reaction with the mineral powder, facilitated by the alkaline environment created by calcium carbide slag. The primary hydration products include C-S-H (calcium–silicate–hydrate) and C-A-S-H (calcium–aluminate–silicate–hydrate) gels, as well as tobermorite, leading to a reduction in water content in the lightweight carbonized cured mud. Concurrently, magnesium oxide reacts with water to form Mg(OH)_2_, which subsequently engages in carbonation reactions with CO_2_ foam. Consequently, the water content tends to decrease with increasing magnesium oxide dosing.

At 13% activated magnesium oxide, the water content is relatively low, indicating a more active internal hydration reaction that consumes a larger amount of water. However, when the dosage exceeds 19%, excessive magnesium oxide hampers the completion of carbonation reactions while hydration reactions continue, resulting in a net increase in wastewater within the mud and, ultimately, a decrease in the water content of the lightweight carbonized cured mud.

#### 3.1.4. Effect of Active Magnesium Oxide Dosage on Unconfined Compressive Strength

**Figure 12:**
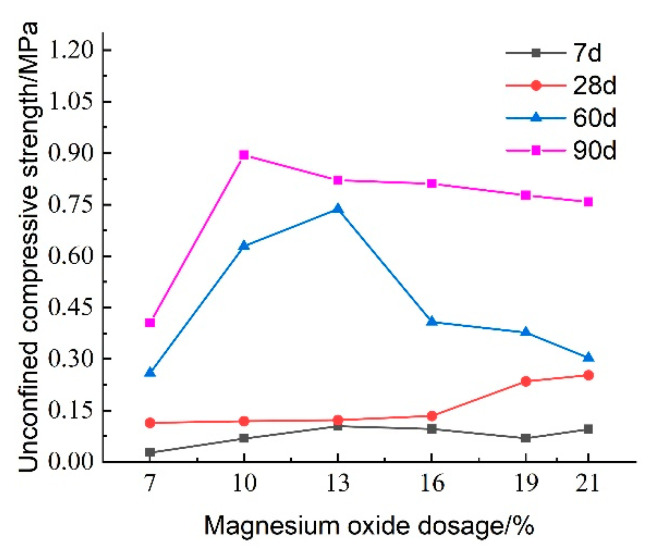
Effect of active magnesium oxide dosage on unconfined compressive strength.

Figure 12 illustrates the effect of reactive magnesium oxide (MgO) content on the unconfined compressive strength of lightweight carbonated solidified slurry. From the figure, it is evident that the MgO content significantly influences the compressive strength. At a curing age of 7 days, the compressive strength of the lightweight carbonated solidified slurry ranges from 0.027 to 0.104 MPa, showing an initial increase followed by a decrease as the MgO content increases. At a curing age of 28 days, the compressive strength ranges from 0.114 to 0.253 MPa, with a continuous increase in strength as the MgO content rises. The maximum compressive strength of 0.253 MPa is achieved when the MgO content is 21%. At longer curing ages of 60 and 90 days, the compressive strength increases rapidly, again showing an initial increase followed by a decrease with increasing MgO content.

As the content of reactive magnesium oxide (MgO) increases, the hydration reaction intensifies, leading to a higher content of hydration products such as Mg(OH)_2_. Subsequently, under high-concentration CO_2_ foam conditions, a reaction occurs, producing granular magnesite crystals. These crystals help fill the pores in the lightweight carbonated solidified slurry and aggregate the slurry particles through encapsulation. At the same time, as the MgO content increases, the number of internal pores in the slurry decreases, and its structure becomes more compact. However, when the MgO content becomes too high, excessive carbonation products cover the unreacted raw materials, preventing the hydration and carbonation reactions from fully occurring, which results in a decrease in the unconfined compressive strength of the lightweight carbonated solidified slurry.

**Figure 13:**
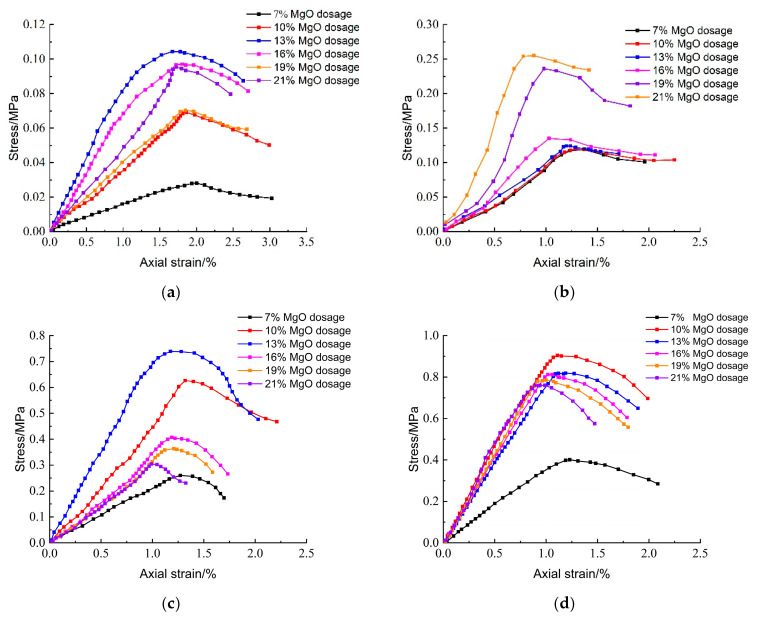
Stress–strain curves at different ages with different active MgO dosages. (**a**) Conservation age 7 d; (**b**) conservation age 28 d; (**c**) maintenance age of 60 d; and (**d**) maintenance age of 90 d.

Figure 13 presents the effect of activated magnesium oxide dosing on the stress–strain behavior of lightweight carbonized cured mud at different maintenance ages. The stress damage can be categorized into three stages: (1) Linear Stage: the specimen experiences a linear increase in axial stress with axial strain up to its peak stress, exhibiting elastic deformation without internal damage or cracks; (2) Nonlinear Stage: the axial stress increases nonlinearly with strain until reaching peak stress, at which point plastic deformation and crack formation begin; and (3) Strain-Softening Stage: after reaching peak stress, further increases in axial strain lead to a gradual reduction in stress as internal cracks expand, culminating in specimen failure.

At 7 days, most specimens show a clear stress peak, especially at 13% MgO dosage, indicating typical strain-softening behavior at early curing stages. At 60 days, all specimens with activated magnesium oxide demonstrate distinct stress peaks, with the highest peak also observed at 13%. Higher dosages enhance the hydration reaction of magnesium oxide, generating Mg(OH)_2_, which coats the magnesium oxide surface, inhibiting further hydration and carbonation reactions. This limits the availability of Mg^2+^ and OH^−^ ions, which are essential for the hydration process, resulting in decreased unconfined compressive strength. At 90 days, the peak stress significantly increases, indicating that the lightweight carbonation-cured mud exhibits brittle behavior prior to failure.

The lack of significant strength increase between day 3 and day 7 may be attributed to the early densification of the matrix through rapid carbonation reactions, which hinder further CO_2_ penetration and reduce reactivity beyond the initial phase. Moreover, as water evaporates under CO_2_-rich curing, further hydration of GGBS and slag components becomes limited. This phenomenon has been reported in other CO_2_-cured systems where early carbonation forms a diffusion barrier on particle surfaces, thus suppressing strength gain after a certain point.

Figure 13b shows typical stress–strain curves for different MgO dosages (7 days). The presence of initial stress offset in some curves is due to minor equipment pre-contact or alignment load during test setup. This offset was considered during analysis.

**Figure 14:**
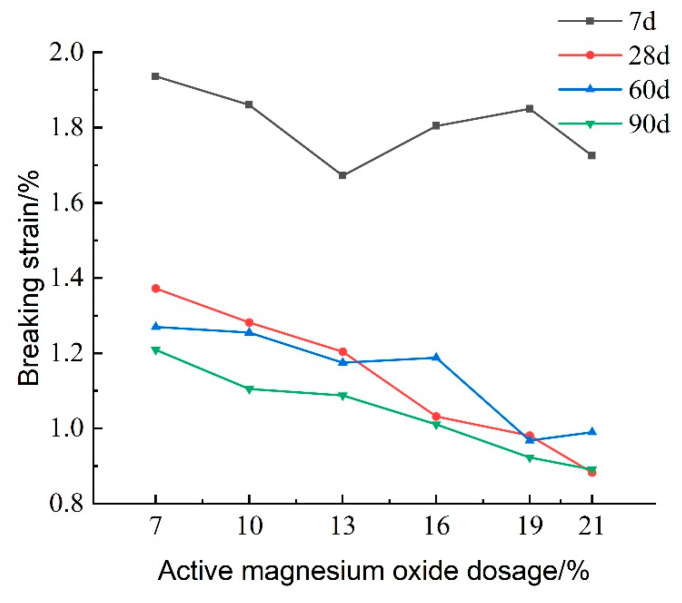
Effect of active magnesium oxide doping on destructive strain.

Destructive strain refers to the strain value corresponding to the peak stress of the lightweight carbonated cured mud and serves as an indicator of the material’s brittleness. Figure 14 illustrates the effect of activated magnesium oxide dosing on the destructive strain of lightweight carbonized cured mud at different maintenance ages. The figure shows that, as the dosage of activated magnesium oxide increases, the destructive strain gradually decreases. Additionally, with longer maintenance periods, the destructive strain continues to decline, indicating that the brittleness of the lightweight carbonized cured mud increases with both higher activated magnesium oxide dosing and longer maintenance time, while its plastic characteristics diminish. At a 7% activated magnesium oxide dosage and a maintenance age of 7 days, the highest destructive strain recorded is 1.936%. This decrease in destructive strain over time can be attributed to the more complete carbonation reaction in the lightweight carbonated cured slurry.

**Figure 15:**
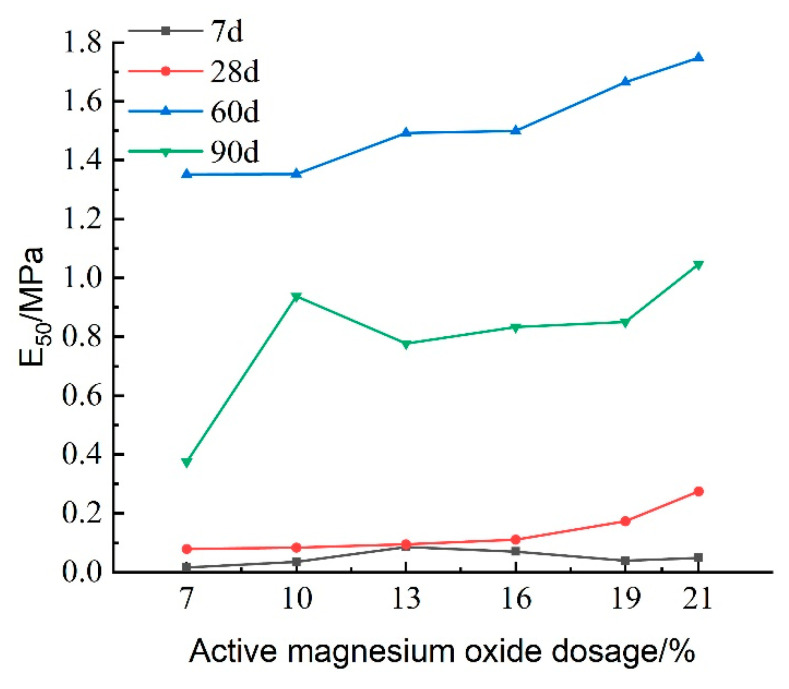
Effect of active magnesium oxide dosing on E_50_.

The deformation modulus (E_50_) of the cured soil is defined as the ratio of 50% of the peak stress to its corresponding strain, reflecting the soil’s ability to resist elastic–plastic deformation. Figure 15 presents the variation in E_50_ with respect to the activated magnesium oxide dosing at different maintenance ages. The figure indicates that E_50_ gradually increases with higher dosages of activated magnesium oxide, suggesting that the internal reactions within the lightweight carbonation-cured mud become more pronounced, leading to an increase in carbonation products and enhanced resistance to deformation. At longer maintenance ages of 60 and 90 days, E_50_ increases more rapidly, indicating that the strength of the lightweight carbonized cured mud improves significantly in the later stages compared to the earlier stages, thereby enhancing its resistance to deformation.

**Figure 16:**
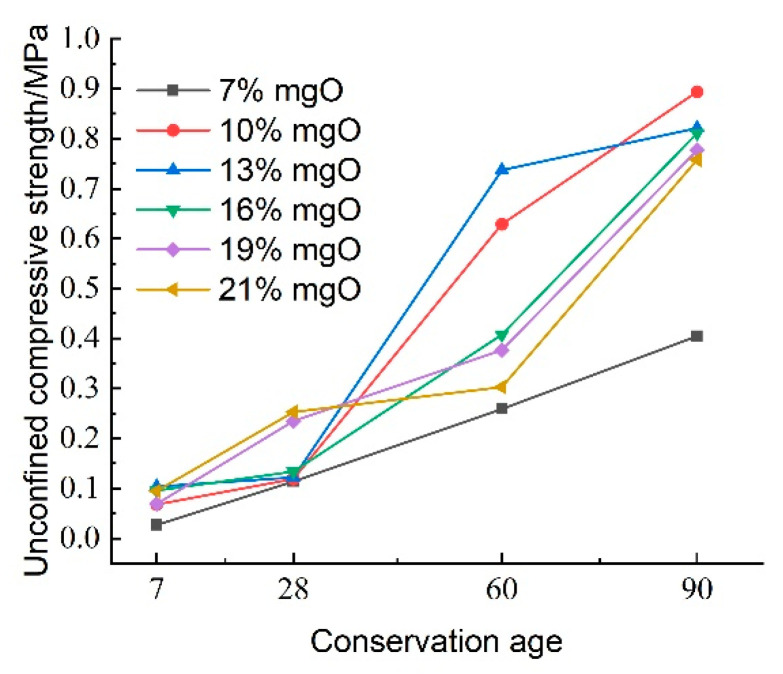
Effect of magnesium oxide dosing on unconfined compressive strength at different ages of maintenance.

Figure 16 illustrates the effect of activated magnesium oxide dosing on the compressive strength of lightweight carbonized cured mud at various maintenance ages. The data indicate that the overall unconfined compressive strength of the lightweight carbonized cured mud increases with curing age. Between maintenance ages of 7 and 28 days, the growth in compressive strength is gradual; however, at activated magnesium oxide dosages of 19% and 21%, the rate of strength increase accelerates. This rapid growth during the shorter maintenance period can be attributed to enhanced internal hydration and carbonation reactions facilitated by the higher activated magnesium oxide dosing, as well as the alkaline environment, which promotes the formation of carbonation products, which effectively fill the internal pores of the specimens.

From 28 to 60 days, the compressive strength of the lightweight carbonized cured mud grows more rapidly, with the highest growth rate observed at an activated magnesium oxide dosage of 13%. For longer maintenance ages of 60 to 90 days, the compressive strength is maximized with 10% and 13% activated magnesium oxide dosing, indicating that strength increases more significantly in the later stages of curing.

Previous studies have shown that optimal carbonation occurs in indoor carbonation chambers, with peak strength achieved at a carbonation time of 6 h. Once this optimal time is reached, the voids in the soil are filled with carbonation products, tightly wrapping the soil particles and hindering further contact with CO_2_, which can limit additional carbonation reactions. Under continuous high pressure, the soil structure may be compromised, leading to a decline in strength. In contrast, this study employs the CO_2_ foaming method to facilitate thorough carbonation, allowing the reaction to extend beyond the optimal carbonation time. Consequently, as maintenance age increases, the compressive strength of the lightweight carbonized cured mud continues to improve.

### 3.2. Effect of CO_2_ Foam Dosing

In this section, waste mud was used as the research object, and CO_2_ foam doping was 36%, 40%, 44%, 48%, and 52% of the total volume of lightweight carbonated cured mud for the experimental study. Physical and mechanical property tests such as wet weight, flow, moisture content, and unconfined compressive strength were carried out to investigate the effect of CO_2_ foam dosing on lightweight carbonation-cured mud.

#### 3.2.1. Effect of CO_2_ Foam Dosing on Wet Weights

**Figure 17:**
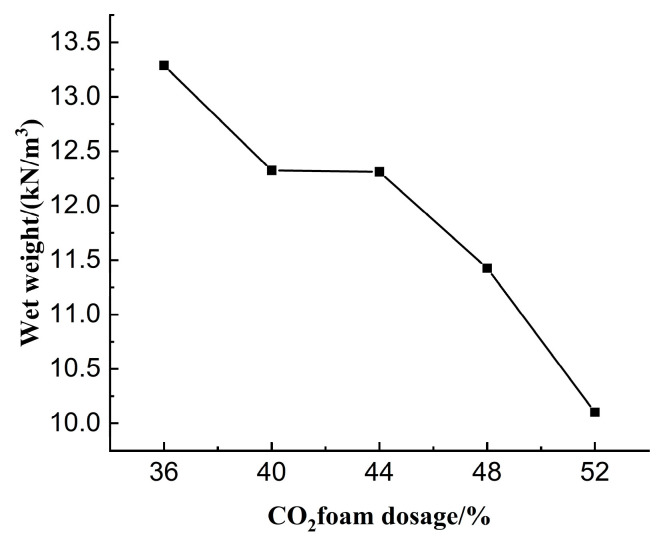
Effect of CO_2_ foam dosing on wet weights.

The experimental results regarding the effect of CO_2_ foam dosage on the wet weight of lightweight carbonated cured mud are presented in Figure 17. The figure illustrates that as the dosage of CO_2_ foam increases, the wet weight of the lightweight carbonized cured mud decreases. Notably, at a foam dosage of 52%, the wet weight reaches a minimum value of 10.103 kN/m^3^, which is lower than that of the cured mud prior to carbonation treatment. Between 40% and 44% CO_2_ foam dosage, the increased availability of CO_2_ creates an optimal environment for internal carbonation reactions within the specimen. This enhances the rate of the carbonation reaction, resulting in an increase in carbonation products and, consequently, a slower rate of reduction in the wet weight of the lightweight carbonated cured mud. Activated magnesium oxide mixed into the slurry rapidly hydrates with the water, generating Mg(OH)_2_, which exists in the form of Mg^2+^ and OH^−^ ions. Mg(OH)_2_ serves as a primary raw material for the carbonation reaction, yielding direct carbonation products such as MgCO_3_ and MgCO_3_·3H_2_O. Additionally, the mineral powder, under the alkaline excitation of calcium carbide slag, releases Mg^2+^ and Ca^2+^ ions, facilitating further hydration and carbonation reactions that produce high-strength carbonates. The specific reaction processes are detailed in Equations (8) and (9).

(9) $$M_{g}{\left(OH\right)}_{2}+{CO}_{2}{\to M}_{g}{CO}_{3}+H_{2}O$$

In the case of a constant amount of curing agent, the wet weight of lightweight carbonized cured mud is primarily influenced by the dosage of CO_2_ foam. The foam structure and the viscosity of the liquid film can impede the flow of the mixture. As the dosage of foam increases, the lightweight carbonized cured mud develops a greater pore space while maintaining the same volume, resulting in a lower mass. This reduction in weight achieves the desired lightweight characteristics, making it suitable for applications such as backfilling after tunnel excavation and foundation filling.

#### 3.2.2. Effect of CO_2_ Foam Dosage on Flowability

**Figure 18:**
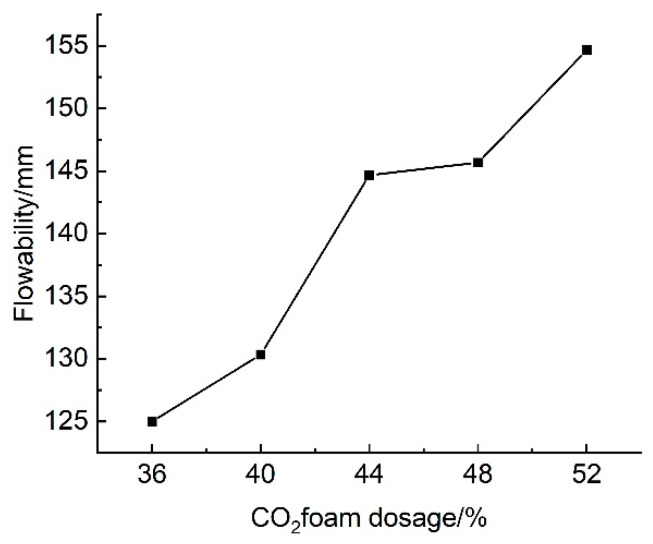
Effect of CO_2_ foam dosing on flowability.

The results of tests on the effect of CO_2_ foam dosage on the flowability of lightweight carbonated cured slurries are illustrated in Figure 18. This figure indicates that, at CO_2_ foam dosages ranging from 36% to 52%, the flow of the lightweight carbonated cured slurry varies between 125 mm and 154.67 mm, which is significantly lower than that of the uncured slurry. Overall, the flow of the lightweight carbonated cured slurry increases with higher CO_2_ foam dosages, reaching a maximum of 154.67 mm at a dosage of 52%.

The increased CO_2_ foam dosage contributes to a greater pore space within the lightweight carbonized cured mud, resulting in overall structural deterioration and a loosening of the internal structure. As foam dosage rises, the pore volume of the lightweight carbonized cured mud expands, leading to increased porosity and the formation of additional connecting pores. Consequently, the mobility of the lightweight carbonized cured mud improves with higher CO_2_ foam dosages.

#### 3.2.3. Effect of CO_2_ Foam Dosage on Water Content

**Figure 19:**
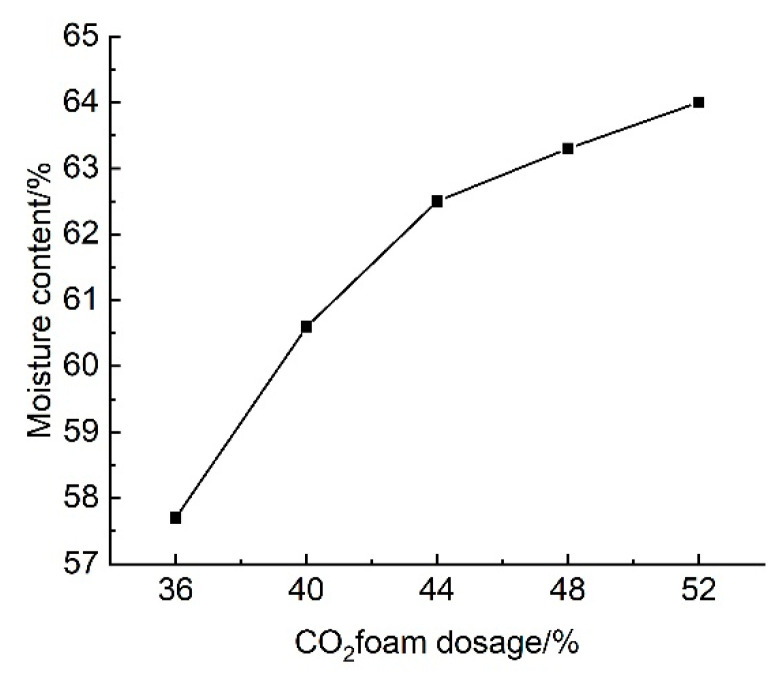
Effect of CO_2_ foam mixing on moisture content.

The results of the tests on the effect of CO_2_ foam dosing on the water content of lightweight carbonated cured slurry are shown in Figure 19.

Figure 19 shows the effect of CO_2_ foam doping on the water content of lightweight carbonation-cured mud; as can be seen from the figure, the water content of lightweight carbonation-cured mud as a whole with the increase in the doping of CO_2_ foam showed an upward trend, and when the doping of CO_2_ foam was 52% it reached a maximum of 64%, compared with the water content of mud without carbonation-cured mud, which reduced by a factor of four.

The change in water content of lightweight carbonation-cured mud can reflect the change in its compressive strength to a certain extent; as the water content of the specimen rises, its internal pores increase, and the structural deterioration and the generation of connecting holes leads to the decrease in the compressive strength of lightweight carbonation-cured mud. At the same time, changes in water content will cause changes in the number of seepage channels in the lightweight carbonation-cured mud, thus affecting the strength of its permeability; the higher the water content, the greater the permeability. Therefore, when selecting the curing agent for the carbonation curing waste mud, the influence of different factors on the water content of lightweight carbonation curing mud needs to be considered.

#### 3.2.4. Effect of CO_2_ Foam Dosing on Unconfined Compressive Strength

**Figure 20:**
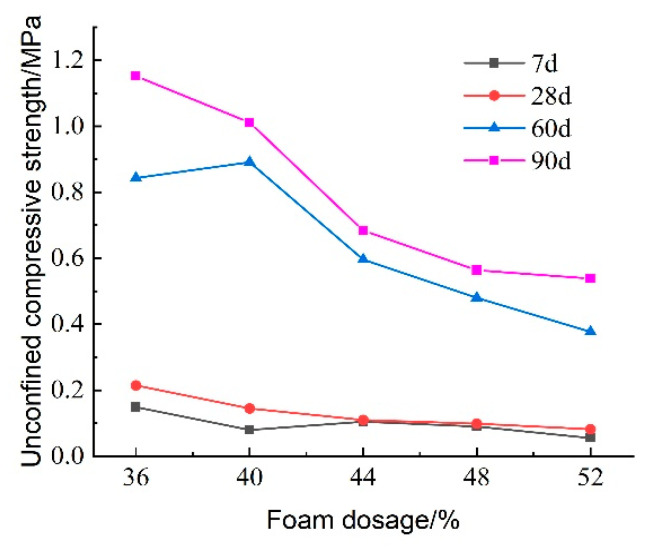
Effect of CO_2_ foam dosing on unconfined compressive strength.

The test results examining the effect of CO_2_ foam dosage on the unconfined compressive strength of lightweight carbonated cured mud at various ages are presented in Figure 20. This figure illustrates that the overall compressive strength of lightweight carbonation-cured mud tends to decrease with increasing CO_2_ foam dosage. The highest compressive strength of 0.215 MPa is observed at a minimum foam dosage of 36% after a maintenance period of 28 days. For shorter maintenance periods of 7 days and 28 days, changes in compressive strength with varying CO_2_ foam dosage are not significant. The incorporation of foam introduces a substantial number of macropores, which increases the pore volume within the lightweight carbonation-cured mud structure, thereby reducing its compressive strength. However, the increased CO_2_ concentration due to foam incorporation enhances the carbonation curing effect, mitigating the overall impact of the pores on compressive strength. As the maintenance period extends, the strength of lightweight carbonation-cured mud increases more significantly. At 90 days of maintenance, the compressive strength is six times greater than that at 28 days, indicating a substantial enhancement in strength over time.

**Figure 21:**
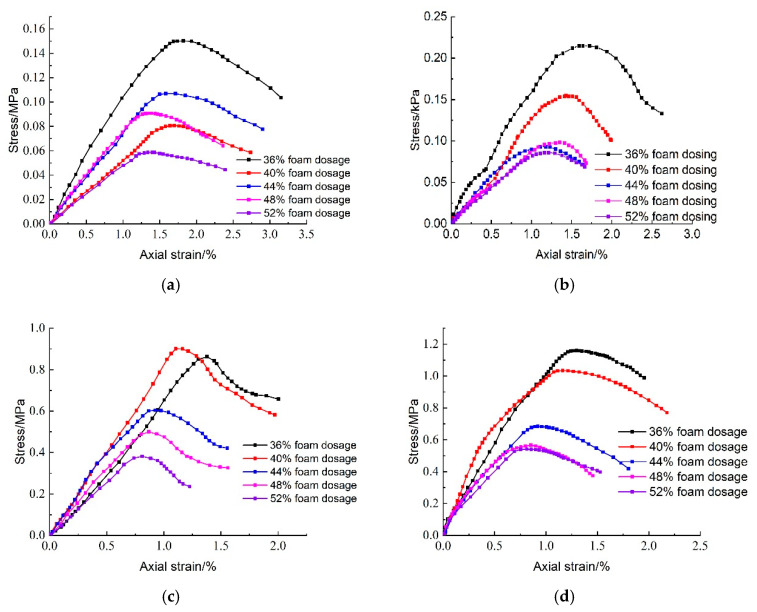
Stress–strain curves at different ages with different CO_2_ foam dosages. (**a**) Age of conservation 7 d; (**b**) age of conservation 28 d; (**c**) conservation age of 60 d; and (**d**) conservation age of 90 d.

Figure 21 illustrates the effect of CO_2_ foam dosage on the stress–strain behavior of lightweight carbonated cured mud under unconfined compressive strength at different maintenance ages. The figure reveals that the stress damage behavior of lightweight carbonated cured mud exhibits a strain-softening pattern. As the specimen reaches peak stress, an increase in axial strain leads to a decrease in stress. Notably, the peak stress of lightweight carbonated cured mud gradually increases with longer maintenance periods. At lower levels of CO_2_ foam doping, the stress peak is pronounced, and significant residual strength is observed. However, as CO_2_ foam dosage increases, the peak stress of the lightweight carbonated cured mud diminishes, and the stress–strain curve flattens. This trend is attributed to the increase in internal pore volume and the resulting decrease in density, which ultimately reduces the strength of the specimen.

**Figure 22:**
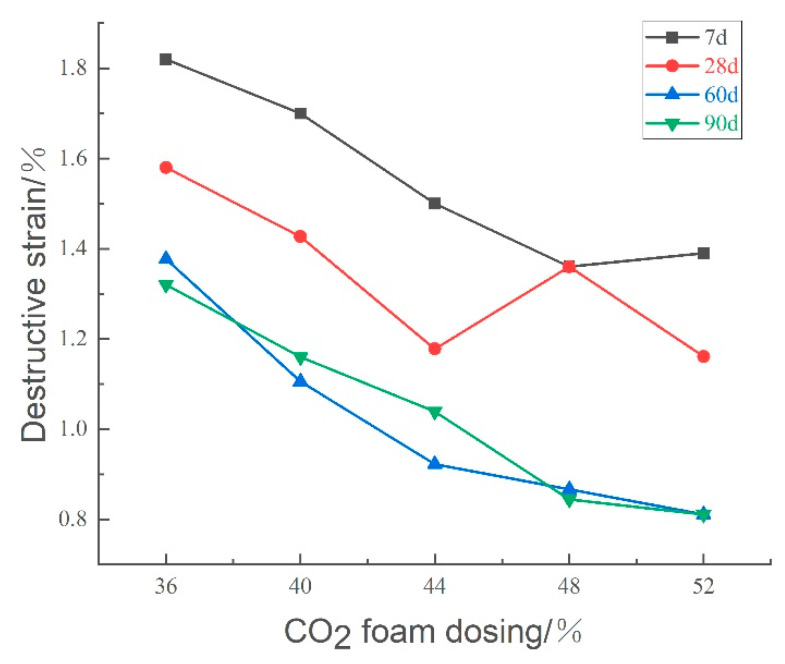
Effect of CO_2_ foam doping on destructive strain.

Figure 22 illustrates the effect of CO_2_ foam dosage on the destructive strain of lightweight carbonated cured mud at various ages. The data indicate a general decreasing trend in destructive strain with increased CO_2_ foam doping and longer maintenance periods. This reduction is attributed to the increase in internal pores resulting from higher foam dosages, which deteriorates the structural properties of the specimen, leading to diminished damaging strain and increased brittleness.

**Figure 23:**
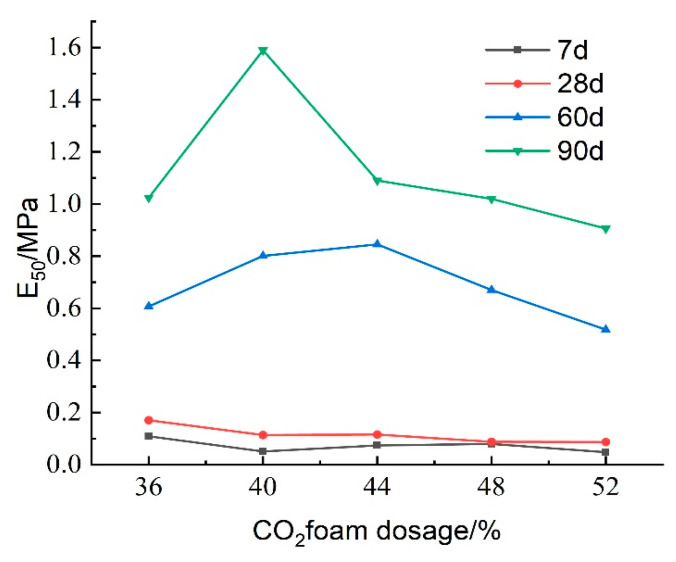
Effect of CO_2_ foam dosing on E_50_.

Figure 23 presents the impact of CO_2_ foam dosing on the deformation modulus (E_50_) of lightweight carbonated cured mud at different maintenance ages. For maintenance periods of 7 and 28 days, E_50_ exhibits a decreasing trend with increasing CO_2_ foam dosage, peaking at 36% foam doping. This trend can be explained by the increased internal porosity and structural deterioration; under the same stress, the specimen experiences greater deformation, resulting in reduced resistance to deformation. In contrast, at longer maintenance ages of 60 and 90 days, E_50_ initially increases and then decreases with higher CO_2_ foam doping. This behavior is due to a more complete carbonation curing reaction over time, which enhances specimen strength and resistance to deformation. The increase in CO_2_ foam doping promotes a favorable carbonation reaction, thereby improving the deformation resistance of lightweight carbonated cured mud to some extent.

**Figure 24:**
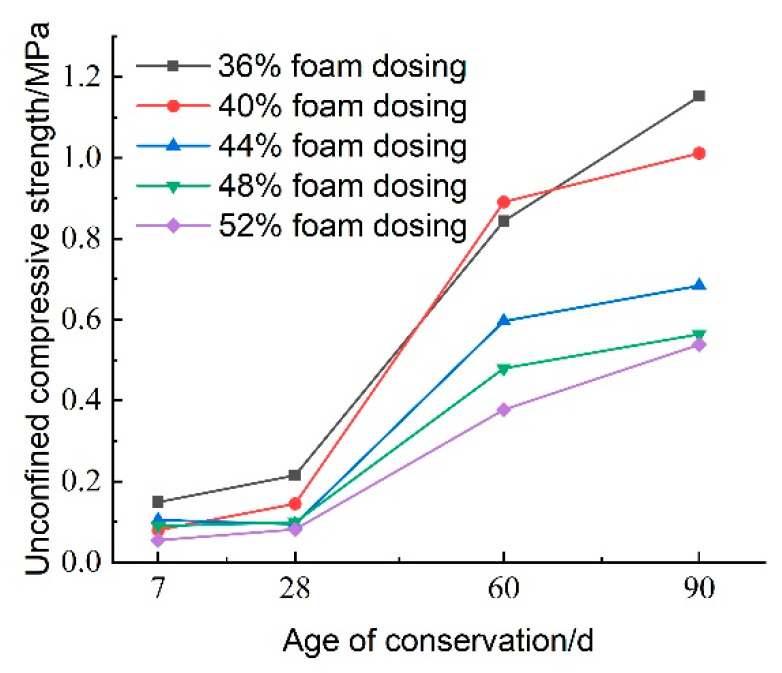
Effect of CO_2_ foam dosing on unconfined compressive strength at different ages of curing.

Figure 24 illustrates the effect of CO_2_ foam dosage on the unconfined compressive strength of lightweight carbonated cured mud at various maintenance ages. The data indicate a general increasing trend in unconfined compressive strength with longer maintenance periods. During the maintenance ages of 7 to 28 days, the growth rate of compressive strength is relatively slow, particularly when CO_2_ foam doping is below 36%. However, from 28 to 60 days, the growth rate accelerates significantly, with specimens exhibiting the highest growth rate at 40% CO_2_ foam doping. Notably, the compressive strength at 60 days is 6.145 times greater than that at 28 days.

While increased CO_2_ foam doping enhances the lightweight properties of the material by increasing internal porosity, it also contributes to overall structural deterioration, resulting in reduced compressive strength. Conversely, as maintenance age increases, the internal carbonation reactions become more complete, leading to an increase in carbonation products that enhance the internal structure.

At 90 days, the unconfined compressive strength of the lightweight carbonated cured mud continues to rise; at 36% CO_2_ foam doping, the compressive strength reaches 1.152 MPa. This finding indicates that the strength of lightweight carbonated cured mud grows more rapidly and to a greater extent in the later stages, consistent with previous test results.

It is noted that for certain high foam dosages (e.g., >48%), the 7-day unconfined compressive strength decreases compared to the low foam dosages. This unfavorable effect can be attributed to the excessive introduction of porosity, which reduces the number of contact points between reactive particles and weakens the skeleton structure of the hardened slurry. Moreover, the increased gas–solid interfaces may slow down carbonation efficiency and reduce curing uniformity. As a result, while low to moderate CO_2_ foam contents (36–44%) enhance early strength by promoting dispersion and internal curing, excessively high contents disrupt structural integrity and delay matrix densification.

### 3.3. Effect of Curing Agent Dosage

This section focuses on the study of waste slurry as the primary material. The curing agent was mixed in proportions of 35%, 40%, 45%, and 50% of the total mass of the waste slurry. The main components of the curing agent included activated magnesium oxide, calcium carbide slag, and mineral powder. Based on results from prior orthogonal tests, the proportions of these components in relation to the total mass of the curing agent were established as follows: 27.8% activated magnesium oxide, 16.7% calcium carbide slag, and 55.5% mineral powder.

Additionally, CO_2_ foam was dosed at 48% of the total volume of the lightweight carbonated cured mud. Anhydrous calcium sulfate was incorporated at 5% of the total mass of the waste slurry, while polyacrylamide (PAM) was added at 0.5% of the total mass. To investigate the effects of curing agent dosing on lightweight carbonated cured mud, a series of physico-mechanical tests were conducted, including assessments of wet weight, fluidity, and unconfined compressive strength.

#### 3.3.1. Effect of Curing Agent Dosage on the Wet Weight of

**Figure 25:**
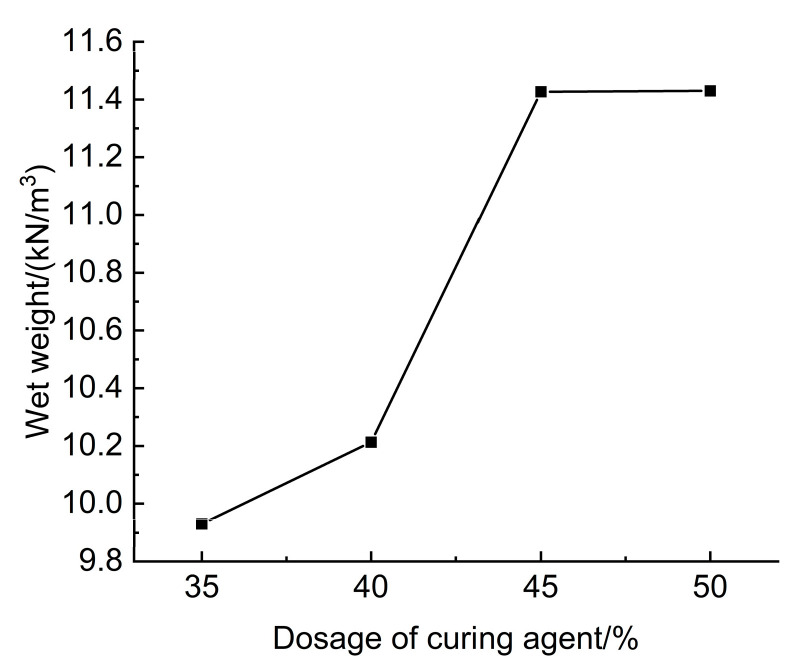
Effect of curing agent dosage on wet weight.

The results of tests assessing the effect of curing agent dosage on the wet weight of lightweight carbonated cured mud are presented in Figure 25. The figure illustrates that when the curing agent dosage ranges from 35% to 50%, the wet weight of the lightweight carbonation-cured mud varies between 9.93 and 11.43 kN/m^3^, with an increase in wet weight corresponding to higher curing agent dosages at a constant CO_2_ foam dosage.

This trend can be attributed to the greater proportion of mineral powder in the curing agent, which has a higher density compared to the waste slurry. Consequently, the density of carbonation curing products, such as magnesium carbonate, is also elevated. As the amount of curing agent increases, the quantity of mineral powder and carbonation products rises, resulting in a higher wet weight for the lightweight carbonation-cured slurry. Notably, the wet weight of the untreated waste slurry is 11.8 kN/m^3^, which exceeds that of the lightweight carbonation-cured mud.

Since the densities of both the curing agent and the carbonation products are greater than that of the waste slurry, it is evident that lightweight characteristics of the carbonation-cured slurry can be achieved by adjusting the curing agent dosage. Furthermore, the mixing amount of CO_2_ foam can also be modified to meet the engineering construction and strength requirements, ensuring both strength and performance while promoting lightweight properties.

#### 3.3.2. Effect of Curing Agent Dosage on Flowability

**Figure 26:**
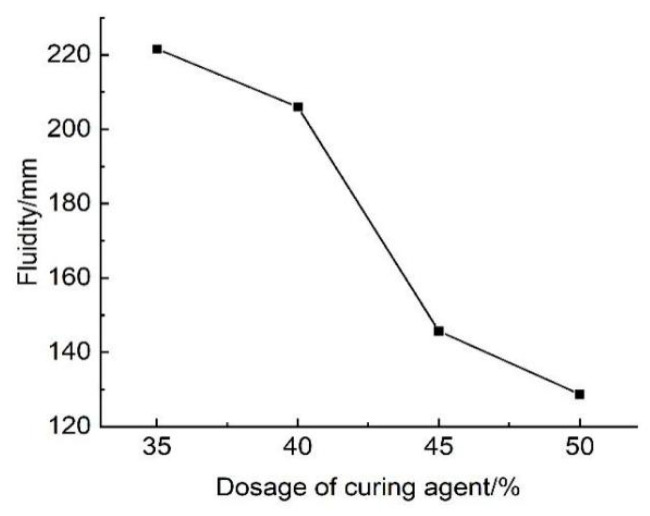
Effect of curing agent dosage on flowability.

The results of tests examining the effect of curing agent dosage on the flowability of lightweight carbonated cured mud are presented in Figure 26. The figure illustrates how the fluidity of the lightweight carbonation curing slurry varies with different curing agent dosages. It can be observed that when the curing agent dosage ranges from 35% to 50%, the fluidity of the lightweight carbonation curing slurry varies between 128 mm and 221 mm. Notably, at a curing agent dosage of 50%, the fluidity drops to 128 mm, which does not meet construction requirements. Therefore, for applications where higher flowability is necessary, it is recommended to use lightweight carbonation curing slurry with a curing agent dosage below 50%.

The decrease in fluidity with increasing curing agent dosage can be attributed to the rising number of soil and curing agent particles in the waste slurry. As the curing agent dosage increases, these particles require a greater amount of free water to remain adequately wet and dissolved. Additionally, the strong adhesion between the wet soil particles and curing agent particles reduces the availability of free water, resulting in increased viscosity and deteriorated construction performance. Moreover, the increase in carbonation curing products with higher curing agent dosages leads to a greater solid content within the samples at the same water content, further contributing to the decrease in fluidity.

#### 3.3.3. Effect of Curing Agent Dosage on Unconfined Compressive Strength

**Figure 27:**
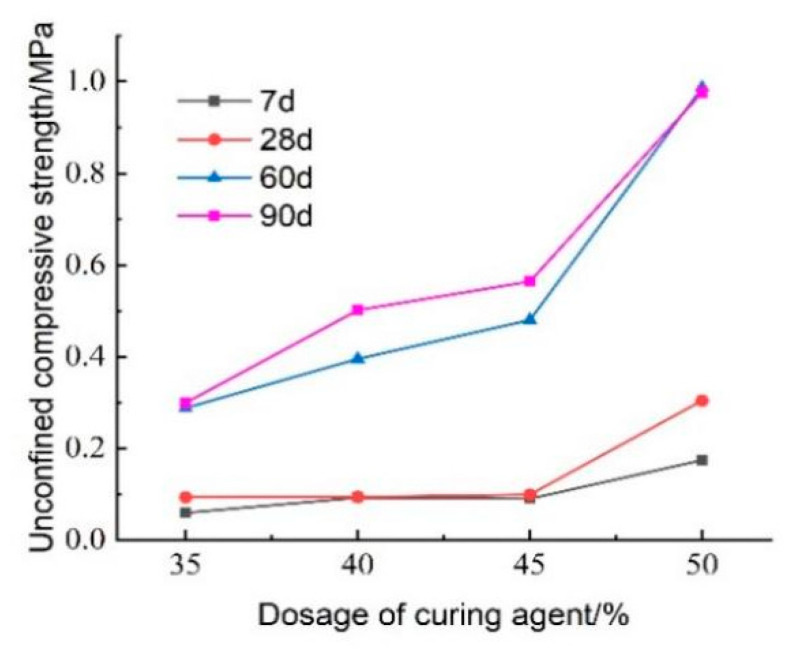
Effect of curing agent dosage on unconfined compressive strength.

The test results regarding the effect of curing agent dosage on the unconfined compressive strength of lightweight carbonated cured mud at various maintenance ages are presented in Figure 27. The figure illustrates that the unconfined compressive strength of lightweight carbonized cured mud generally increases with higher curing agent dosages, reaching its maximum at a dosage of 50%. At lower curing agent dosages of 35–45%, the influence on compressive strength is minimal. Specifically, at a maintenance age of 28 days, the compressive strength of lightweight carbonation-cured mud with a 50% curing agent dosage can achieve 0.304 MPa. This enhancement in strength is attributed to the increased dosage facilitating more complete hydration and carbonation reactions within the lightweight carbonation-cured mud. Additionally, the substantial formation of carbonation products helps fill the sample’s pores, reinforcing its structure and significantly contributing to the overall strength of the specimen. This structural enhancement is a crucial factor in optimizing curing agent dosage.

**Figure 28:**
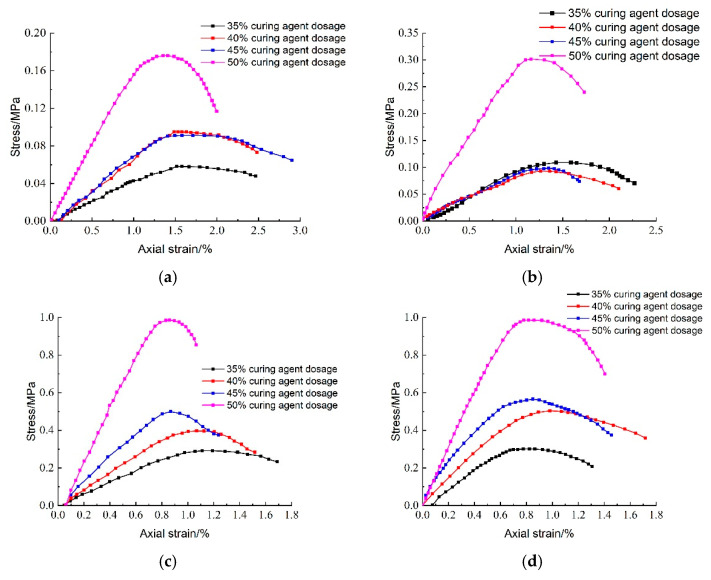
Stress–strain curves at different ages with different curing agent dosages. (**a**) Conservation age of 7 d; (**b**) conservation age of 28 d; (**c**) maintenance age of 60 d; and (**d**) maintenance age of 90 d.

Figure 28 illustrates the effect of hardener dosing on the stress–strain behavior of lightweight carbonized cured mud at various maintenance ages. The data reveal a significant increase in peak stress as hardener dosage rises, with the highest peak stress observed at a hardener doping level of 50%. This enhancement is attributed to the increased availability of raw materials for the carbonation curing reaction within the specimen, leading to a greater generation of carbonation products. Additionally, as maintenance age increases, the peak stress of the lightweight carbonation-cured mud also rises, further demonstrating the positive impact of both hardener dosage and aging on the material’s mechanical properties.

**Figure 29:**
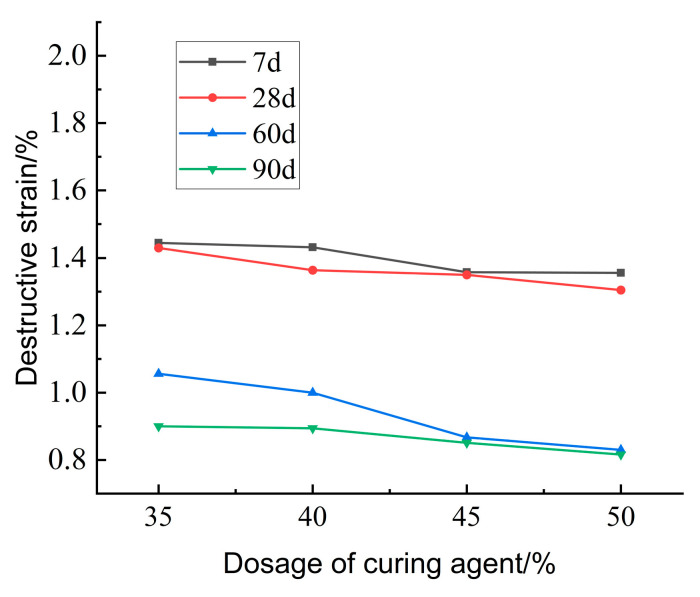
Effect of curing agent dosage on destructive strain.

Figure 29 illustrates the effect of curing agent dosage on the destructive strain of lightweight carbonized cured mud at various ages. The data indicate that as both curing agent dosage and maintenance age increase, the destructive strain of the lightweight carbonation-cured mud decreases. This trend suggests a rise in brittleness, with the damage mechanism shifting toward more brittle failure modes. The increased curing agent dosage leads to a higher volume of internal carbonation products, resulting in a denser structure and enhanced brittleness and hardness of the specimen.

**Figure 30:**
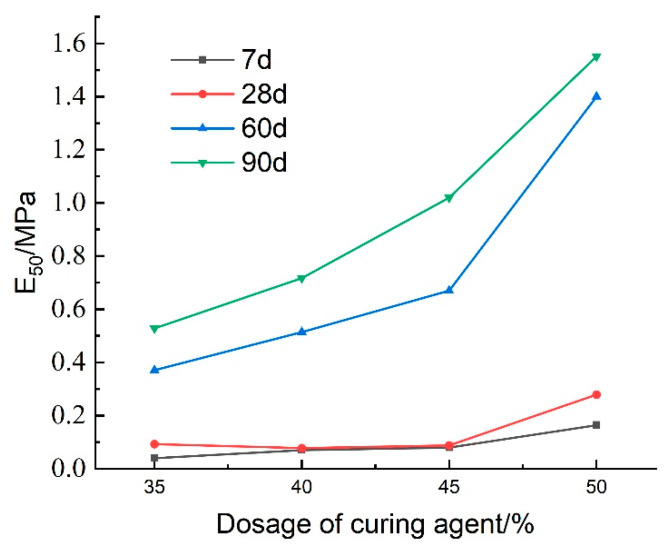
Effect of curing agent dosage on E_50_.

Figure 30 presents the effect of curing agent dosage on the deformation modulus E_50_ of lightweight carbonation-cured mud at different ages. The results show that E_50_ increases with both curing agent dosage and curing age, with higher values observed at maintenance ages of 60 days and 90 days. This increase is attributed to more complete internal carbonation and curing reactions over time, which enhance the specimen’s strength and structural integrity, thereby improving the deformation resistance of the lightweight carbonation-cured mud.

**Figure 31:**
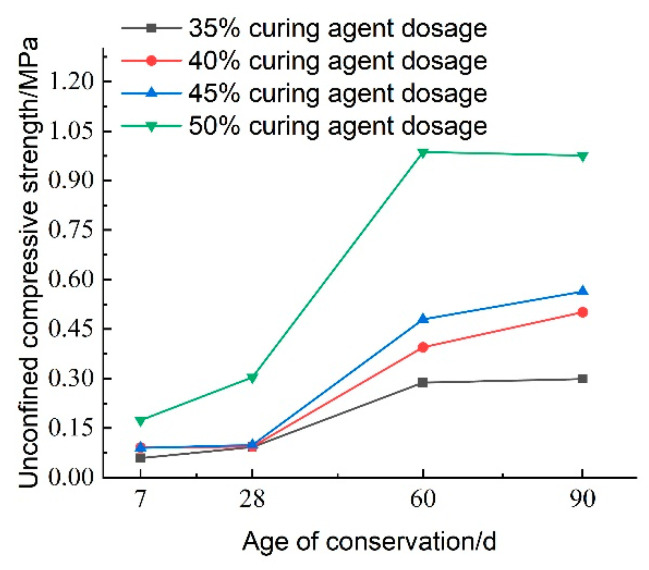
Effect of curing agent dosage on unconfined compressive strength at different curing ages.

Figure 31 illustrates the effect of curing agent dosage on the unconfined compressive strength of lightweight carbonized cured mud at various maintenance ages. The data indicate that maintenance age significantly influences compressive strength, which exhibits an increasing trend over time. Between 7 and 28 days, the compressive strength of the lightweight carbonized cured mud increases slowly with the addition of the curing agent, with the strength at 28 days approximately 1.1 times that at 7 days. In contrast, from 28 to 60 days, the compressive strength experiences a more rapid increase, reaching about three times the strength at 28 days, particularly when the curing agent dosage is at 50%.

From 60 to 90 days, the strength continues to grow, albeit at a slower rate, indicating that the internal carbonation reactions are not yet fully complete. This suggests that the material has significant late-strength potential, consistent with previous findings showing that the late strength of lightweight carbonized cured mud exceeds the early strength. Consequently, this material is suitable for applications requiring lower early strength, such as tunneling and excavation backfill for retaining walls.

In contrast to the strength stagnation observed in high-foam-content mixtures (Section 3.2.4), the continuous strength gain at 7 days with increasing curing agent dosage can be attributed to the higher availability of reactive binders (MgO and CaO), which enhances both carbonation and hydration reactions. The elevated solid content promotes more complete matrix formation and continuous CO_2_ uptake over the curing period. Unlike foam addition, which introduces structural porosity and may delay strength development, the increased dosage of curing agents contributes to a denser and more cohesive microstructure. This difference highlights the dual role of the curing agent in both initiating early strength and supporting long-term mechanical improvement.

## 4. Discussion

In this study, the dosages of CO_2_ foam (35%) and polyacrylamide (PAM, 0.5%) were selected based on previous research [19,20]; however, their applicability to the specific waste slurry used herein was further evaluated through extended parametric analysis. For CO_2_ foam, a series of tests were conducted with dosages ranging from 36% to 52%, as detailed in Section 3.2. These results indicated that a foam content around 36–40% achieved a balance between compressive strength and flowability, validating the feasibility of the initially adopted 35% dosage. Similarly, the addition of 0.5% PAM demonstrated sufficient flocculation and dewatering capacity when used in conjunction with anhydrous calcium sulfate, in accordance with findings reported by Yang [23]. The curing agent dosage was also systematically varied and its effects evaluated (Section 3.3), further supporting the overall optimization approach. Nonetheless, future work could benefit from a more comprehensive sensitivity analysis across broader slurry compositions to enhance the generalizability of the parameter selections.

### 4.1. Influence of Key Material Dosages on Physical and Mechanical Properties

The impact of active magnesium oxide dosage on wet weight, flow, moisture content, and unconfined compressive strength was also examined. The wet weight increased with active MgO dosage, peaking at 21%. The flowability ranged from 122 to 195 mm, decreasing with higher MgO levels, and maximum flowability occurred at 7%. Water content displayed a fluctuating trend, with the lowest point at 21% MgO. Compressive strength showed an initial increase followed by a decrease at 7 days, peaking at 13%, while at 28 days, the highest strength was observed at 21%. Strength continued to rise significantly with longer maintenance periods. The effect of CO_2_ foam dosage revealed a decrease in wet weight as the dosage increased, reaching a minimum at 52%, which was lower than the weight of the uncarbonated slurry. Flowability increased with CO_2_ foam, peaking at 52%. Water content also increased overall, with the maximum at 52%, significantly lower than that of the uncarbonated slurry. Compressive strength decreased with higher CO_2_ foam dosage, reaching its maximum at 36%, with a notable increase in strength over longer maintenance periods. The analysis of curing agent dosage demonstrated that wet weight ranged from 9.93 to 11.43 kN/m^3^ at 35% to 50% dosage, consistently increasing and remaining lighter than the untreated slurry. Flowability ranged from 128 to 221 mm, decreasing with increased curing agent dosage, with 50% not meeting construction requirements. Compressive strength rose with increased dosage, peaking at 50%, and maintenance age significantly influenced strength, with later stages exhibiting much higher values, making it suitable for projects with lower early strength requirements.

### 4.2. Trend Projections Beyond Tested Ranges

In this study, the maximum unconfined compressive strength for MgO-doped specimens was observed at the highest tested dosage of 21%. While this suggests potential for further strength gains at higher MgO contents, it is also important to note that destructive strain and flowability decreased sharply with increasing MgO, indicating an unfavorable increase in brittleness. Based on these trends, it is hypothesized that MgO dosages exceeding 21% may continue to improve early-age strength but with diminishing returns and significant penalties in workability and ductility.

Similarly, the CO_2_ foam dosage tests indicated that flowability continued to increase up to the maximum tested value of 52%, while compressive strength gradually declined. Given the rapid increase in porosity and water content at high foam dosages, it is likely that dosages beyond 52% would lead to structurally unstable mixtures with poor mechanical performance, even if lightweight characteristics are enhanced. Future studies should explore these extended ranges with caution, preferably using multi-objective optimization (e.g., strength vs. weight vs. flowability) to define practical upper limits.

### 4.3. Flowability Criteria and Constructability Implications

The flowability of lightweight carbonated cured slurry is a key parameter affecting its pumpability, mixing uniformity, and placement efficiency in field engineering. According to the *Technical Specification for Filling Engineering with Bubble Mixed Lightweight Soil* (CJJ/T177-2012), the recommended flowability range for construction-grade bubble-mixed lightweight soil is 160–200 mm. This range ensures that the material can be effectively pumped and uniformly placed while maintaining sufficient foam stability and strength development.

In this study, the flowability of some mixtures—particularly those with higher curing agent dosages (e.g., 50%)—was recorded as low as 128 mm, which falls below the minimum construction standard. Slurries with such low flowability exhibit increased viscosity and reduced mobility, posing challenges for pipeline transportation and increasing the risk of clogging. Additionally, poor flowability hinders the even distribution of CO_2_ foam and stabilizers within the slurry, potentially leading to an inhomogeneous structure and compromised mechanical performance.

Conversely, flowability values exceeding 200 mm may lead to segregation, rapid foam collapse, or inconsistent solidification. Therefore, based on the experimental results and standard requirements, this study recommends a target flowability range of 160–190 mm to balance constructability, pumpability, and mechanical integrity. Future work should explore rheological behavior under dynamic flow and evaluate full-scale pumping trials to further refine these thresholds.

### 4.4. Trade-Offs Between Strength and Brittleness: Implications and Mitigation

The experimental results demonstrate that increased dosages of reactive magnesium oxide and curing agents significantly improve the compressive strength of lightweight carbonated cured slurry. However, this gain in strength is accompanied by a marked reduction in destructive strain and an increase in the deformation modulus (E_50_), indicating enhanced brittleness. From an engineering standpoint, high brittleness can compromise the ductility and energy absorption capacity of the material under dynamic or differential loading conditions, such as in seismic zones or areas with soft foundation variability.

This strength–brittleness trade-off suggests that while higher MgO dosages improve load-bearing performance, they may reduce the material’s ability to tolerate deformation without cracking. To manage this, potential strategies include the following: blending with ductile materials such as polypropylene fibers or rubber particles to enhance post-peak behavior; optimizing MgO content below critical levels (e.g., 13–16%) where strength gain plateaus while brittleness escalates; and modifying the curing regime, such as staged carbonation or extended humidity-controlled curing, to adjust the microstructure and mitigate excessive stiffness.

Additionally, applications that prioritize compressive capacity over ductility—such as vertical load-bearing fills or static embankments—may be more suitable for high-MgO formulations. In contrast, uses requiring flexural resistance or long-term deformation capacity (e.g., base layers under pavements) may benefit from hybrid formulations.

### 4.5. Environmental Impact and Sustainability Considerations

Although this study primarily focuses on the mechanical and physical performance of lightweight carbonated cured slurry, its development is also motivated by the need for more sustainable construction materials. Compared to Portland cement, reactive magnesium oxide (MgO) can be produced at significantly lower calcination temperatures (~700 °C vs. >1450 °C), leading to reduced energy consumption and lower process emissions. According to Liska et al. [27] and Walling and Provis [28], the CO_2_ emissions of reactive MgO production can be as low as 50–70% of those of traditional cement when using seawater or serpentine-derived sources.

Furthermore, the carbonation process employed in this study utilizes CO_2_ not only as a reaction agent but also as a carbon sequestration mechanism. While the CO_2_ used was high-purity industrial gas, future applications may incorporate flue gas or biogenic CO_2_ streams, improving net carbon balance. The use of industrial by-products such as ground granulated blast furnace slag (GGBS) and calcium carbide slag further contributes to resource circularity.

Overall, although a complete life cycle assessment (LCA) was not conducted, the material system presented here aligns with core sustainability principles: reduced embodied energy, partial CO_2_ sequestration, and utilization of industrial waste. Future work should quantitatively assess the global warming potential (GWP), energy input, and carbon sequestration efficiency across different mix designs and field scales.

### 4.6. Engineering Implications and Scalability

Although this study comprehensively evaluated the physical and mechanical properties of lightweight carbonated cured slurry at the laboratory scale, its practical implementation in field engineering projects requires further consideration. In terms of scalability, several engineering challenges may arise, including the uniform mixing of high-water-content waste slurry with multiple solid powders at larger volumes, the stability of CO_2_ foam under site-specific environmental conditions, and the logistics of on-site carbonation. Additionally, the curing environment (e.g., temperature and humidity control) used in the lab may not be replicable in field conditions, potentially affecting strength development and carbonation efficiency.

Material availability and cost are also key factors in determining field feasibility. While reactive magnesium oxide, ground granulated blast furnace slag (GGBS), and calcium carbide slag are industrial by-products or readily available materials, their local sourcing may affect economic viability. CO_2_ supply and foaming equipment may also require customization for field-scale deployment. Despite these challenges, the lightweight and carbon-sequestering properties of the proposed material suggest promising potential for use in applications such as backfilling, roadbed reinforcement, and soft ground improvement.

Furthermore, the adaptability of this method to various geotechnical conditions—such as cohesive soils, silt, or high-organic-content muds—should be evaluated. Site-specific pilot studies are recommended to verify the performance of the material under realistic stress conditions and environmental variability. Future research should focus on developing mobile mixing and carbonation units and on quantifying long-term durability and environmental impact in real-world settings.

## 5. Conclusions

Based on the optimal mix proportion, this study conducted experiments on the wet density, flowability, moisture content, and unconfined compressive strength of lightweight carbonated solidified slurry. The effects of reactive magnesium oxide (MgO) content, CO_2_ foam dosage, and stabilizer dosage on the physical and mechanical properties of the slurry were investigated, and the following conclusions were drawn:

(1) Increasing active magnesium oxide dosage leads to an increase in wet weight, a decrease in flowability, and a complex trend in moisture content (initial decrease followed by increases). Unconfined compressive strength generally increases with active magnesium oxide, peaking at 10% and 13% for longer maintenance ages (60 and 90 days), with the maximum strength reaching 0.894 MPa. The destructive strain decreases with increasing active magnesium oxide dosage and maintenance age, while the deformation modulus (E50) shows an upward trend.
(2) The dosage of CO_2_ foam significantly impacts the physical and mechanical properties of the slurry. As CO_2_ foam increases, wet weight decreases while fluidity and water content increase. At 36% CO_2_ foam, the wet weight reached 1328.67 × 10^2^ kN/m^3^, still lower than the weight of non-foam carbonated slurry. While early compressive strength is less affected, longer maintenance ages show increased strength. Destructive strain and deformation modulus (E50) trends indicate variability with CO_2_ foam dosage.
(3) Increasing curing agent dosage correlates with a higher wet weight and lower fluidity. At a dosage of 35%, fluidity exceeds 200 mm, while unconfined compressive strength increases with both curing agent dosage and maintenance age. Notably, between 45 and 50% curing agent dosage, strength increases rapidly. The destructive strain decreases with increased curing agent dosage and maintenance age, and the late strength significantly surpasses the early strength, highlighting the influence of maintenance age on mechanical properties.
(4) Based on the comprehensive analysis of mechanical properties, fluidity, and structural characteristics, the optimal dosages for the composite curing system are proposed as follows: 25% ground granulated blast furnace slag (GGBS), 12.5% reactive magnesium oxide (MgO), and 7.5% calcium carbide slag. For the foam component, a CO_2_ foam dosage of 36–40% is recommended to ensure sufficient flowability (≥160 mm) and maintain adequate compressive strength. The overall curing agent dosage is optimized at 30–35% of slurry dry mass, balancing strength gain and workability. This optimized mix design provides a practical basis for field implementation in lightweight filling or ground improvement applications, with potential for CO_2_ utilization and partial carbon sequestration.

## Figures and Tables

**Table 1:** Physico-chemical quality of waste slurry.

Water Content (%)	pH	Liquid Limit (%)	Plastic Limit (%)	Specific Gravity (g/cm^3^)
197.7	7.28	31	18.5	1.18

**Table 2:** Chemical composition of active magnesium oxide.

Ingredient	MgO (%)	CaO (%)	Fe (%)	Al (%)	Hydrochloric Acid Insoluble Matter (%)	Water	Forging Losses
Quantity contained	98.00	0.60	0.02	0.01	0.10	0.5	0.5

**Table 3:** Chemical composition of calcium carbide slag.

Ingredient	Al_2_O_3_ (%)	CaO (%)	Ca(OH)_2_ (%)	SiO_2_ (%)	H_2_O (%)
Quantity contained	1.62	6.56	87.82	3.50	0.50

**Table 4:** Chemical composition of mineral powders.

Ingredient	CaO (%)	SiO_2_ (%)	Al_2_O_3_ (%)	SO_3_ (%)	Fe_2_O_3_ (%)	MgO (%)	MnO (%)	Water (%)
Quantity contained	35.00	33.50	17.50	1.65	1.03	6.01	4.91	0.4

**Table 5:** Table of technical conditions for anhydrous calcium sulfate.

Content of Anhydrous Calcium Sulfate (%)	Ammonium Content (%)	Drying Vector (%)	Hydrochloric Acid Insoluble Matter (%)	Alkali Metals and Magnesium (%)	Forging Losses
97.00	0.03	1.00	0.05	0.50	1.42

**Table 6:** Experimental design table for active magnesium oxide dosing in lightweight carbonated cured muds.

Test No.	Mineral Powder Dosage (%)	MgO Doping (%)	Dosage of Calcium Carbide Slag (%)	CO_2_ Foam Dosage (%)	Age of Conservation (d)
1	25.00	7.00	7.50	50.00	7, 28, 60, 90
2	25.00	10.00	7.50	50.00	7, 28, 60, 90
3	25.00	13.00	7.50	50.00	7, 28, 60, 90
4	25.00	16.00	7.50	50.00	7, 28, 60, 90
5	25.00	19.00	7.50	50.00	7, 28, 60, 90
6	25.00	21.00	7.50	50.00	7, 28, 60, 90

**Table 7:** Experimental design table for CO_2_ foam dosing in lightweight carbonated cured mud.

Test No.	Mineral Powder Dosage (%)	MgO Doping (%)	Dosage of Calcium Carbide Slag (%)	CO_2_ Foam Dosage (%)	Age of Conservation (d)
O (1)	25.00	12.50	7.50	36.00	7, 28, 60, 90
O (2)	25.00	12.50	7.50	40.00	7, 28, 60, 90
O (3)	25.00	12.50	7.50	44.00	7, 28, 60, 90
O (4)	25.00	12.50	7.50	48.00	7, 28, 60, 90
O (5)	25.00	12.50	7.50	52.00	7, 28, 60, 90

**Table 8:** Experimental design of waste slurry curing agent dosage table.

Test No.	Dosage of Curing Agent (%)	Mineral Powder Dosage (%)	MgO Doping (%)	Dosage of Calcium Carbide Slag (%)	CO_2_ Foam Mixing (%)	Age of Conservation (d)
S (1)	35.00	19.44	9.72	5.83	48.00	7, 28, 60, 90
S (2)	40.00	22.20	11.11	6.67	48.00	7, 28, 60, 90
S (3)	45.00	25.00	12.50	7.50	48.00	7, 28, 60, 90
S (4)	50.00	27.78	13.89	8.33	48.00	7, 28, 60, 90

## Data Availability

The original contributions presented in this study are included in the article. Further inquiries can be directed to the corresponding author.
